# Supplementary material for: The effectiveness of a web-based Dutch parenting program to prevent overweight in children 9–13 years of age: Results of a two-armed cluster randomized controlled trial
Source: PLoS One. 2022 Oct 21;17(10):e0276168. doi: 10.1371/journal.pone.0276168 (PMC9586369; doi:10.1371/journal.pone.0276168)
Supplement: S1 Appendix — (DOCX) [file pone.0276168.s001.docx]

**S1 Appendix. Overview of parenting scale**

**Table 1.** Overview of parenting scales

| **Scale** | **N items** | **Cronbach’s alpha^1^** | **Cronbach’s alpha^2^** | **Example of items** |
| --- | --- | --- | --- | --- |
| **General parenting style#^a^** |  |  |  |  |
| Involvement | 9 | 0.79 | - | “My family regularly does things for fun together”. |
| Strictness | 6 | 0.78 | - | “I know exactly what my child does in his/her free time”. |
| **Specific EBRB parenting practice** |  |  |  |  |
| ***Parenting feeding style (monitoring)^b^*** |  |  |  |  |
| Instrumental feeding | 4 | 0.74 | 0.58 | In order to get my child to behave him/herself I promise him/her something to eat. |
| Emotional feeding | 5 | 0.80 | 0.77 | I gave my child something to eat to make him/her feel better when he/she is feeling upset. |
| Encouragement to eat | 8 | 0.75 | 0.78 | I encourage my child to foods that he/she has not tasted before. |
| Control over eating | 10 | 0.72 | 0.71 | I allow my child to choose which foods to have for meal. |
| ***Physical activity health policies (monitoring)^b^*** | 5 | 0.62 | 0.56 | How often do you encourage your child to be physical active? |
| ***Healthy eating role modeling^b^*** | 12 | 0.68 |  | “How often do you drink SSB while your child is around?” |
| ***Sedentary behavior role modeling^b^*** | 2 | 0.41 |  | How often does your child see you watching television? |
| ***Physical activity role modeling^b^*** | 6 | 0.59 |  | How often does your child see you being physically active (e.g. walking, cycling, playing sports)? |
| **Parental self-efficacy^c^** |  |  |  |  |
| Efficacy | 7 | 0.77 |  | If anyone can find the answer to what is troubling my child, I am the one. |
| Satisfaction | 9 | 0.78 |  | Being a parent makes me tense and anxious |

^1^ Baseline questionnaire parents; ^2^ Baseline questionnaire child; ^a^ 5-point Likert scale: completely disagree (-) to completely agree (+2); ^b^ 5-point Likert scale: never (1) to always (5); ^c^ 6-point Likert scale: strongly disagree (1) to strongly agree (6).
