# Supplementary material for: The effectiveness of a web-based Dutch parenting program to prevent overweight in children 9–13 years of age: Results of a two-armed cluster randomized controlled trial
Source: PLoS One. 2022 Oct 21;17(10):e0276168. doi: 10.1371/journal.pone.0276168 (PMC9586369; doi:10.1371/journal.pone.0276168)
Supplement: S2 Appendix — (DOCX) [file pone.0276168.s002.docx]

**S2 Appendix. Demographic and weight-related characteristics of study population (Completers-only)**

**Table 1a.** Demographic and weight-related characteristics of the study population (Completers-only)

|  | **Intervention condition**  **N (%)** | **Control condition**  **N (%)** | **p-value** |
| --- | --- | --- | --- |
| **Child** |  |  |  |
| **Age, years (mean ± SD)** | 10.4 ± 1.1 | 10.4 ± 1.1 | 0.903^a^ |
| **Gender** |  |  |  |
| Male | 87 (53.0) | 95 (53.7) |  |
| Female | 77 (47.0) | 82 (46.3) | 0.908^b^ |
| **Ethnicity** |  |  |  |
| Caucasian | 158 (96.3) | 169 (95.5) |  |
| Non-Caucasian | 6 (3.7) | 8 (4.5) | 0.689^b^ |
| **Weight status based on BMI** |  |  |  |
| Not overweight | 135 (82.3) | 146 (82.5) |  |
| Overweight/obese | 29 (17.7) | 31 (17.5) | 0.967^b^ |
|  |  |  |  |
| **Parent** |  |  |  |
| **Age, years (mean ± SD)** | 43.0 ± 4.8 | 42.5 ± 4.5 | 0.360^a^ |
| **Ethnicity** |  |  |  |
| Caucasian | 155 (95.1) | 170 (96.0) |  |
| Non-Caucasian | 8 (4.9) | 7 (4.0) | 0.677^b^ |
| **Education level** |  |  |  |
| Low | 52 (31.7) | 55 (31.3) |  |
| Middle | 78 (47.6) | 91 (51.7) |  |
| High | 34 (20.7) | 30 (17.0) | 0.634^b^ |
| **Weight status based on BMI** |  |  |  |
| Not overweight | 80 (51.6) | 97 (55.7) |  |
| Overweight/obese | 75 (48.4) | 77 (44.3) | 0.453^b^ |

Unless indicated otherwise, data are presented as N (%).

^a^ Student’s *t*-test; ^b^ Chi-square test
