# Supplementary material for: The effectiveness of a web-based Dutch parenting program to prevent overweight in children 9–13 years of age: Results of a two-armed cluster randomized controlled trial
Source: PLoS One. 2022 Oct 21;17(10):e0276168. doi: 10.1371/journal.pone.0276168 (PMC9586369; doi:10.1371/journal.pone.0276168)
Supplement: S3 Appendix — (DOCX) [file pone.0276168.s003.docx]

**S3 Appendix. Baseline characteristics of children’s EBRB and parenting dimensions (Completers-only)**

**Table 2a.** Baseline Characteristics of the children’s EBRBs and the parenting dimensions and inter-rater reliability (IRR) within n=475 parent-child dyads (Completers-only)

|  | **According to the children** | | **According to the parents** | | **Agreement among answers parent-child** |
| --- | --- | --- | --- | --- | --- |
|  | **Intervention**  **N (%)** | **Control**  **N (%)** | **Intervention**  **N (%)** | **Control**  **N (%)** | **IRR** |
| **Dietary behavior (mean ± SD)** |  |  |  |  |  |
| Days of having breakfast per week | 6.6 ± 1.1 | 6.7 ± 1.0 | 6.8 ± 0.72 | 6.8 ± 0.63 | 0.41 |
| Portions of fruit per week | 15.1 ± 9.2 | 15.0 ± 9.1 | 10.8 ± 6.6 | 11.1 ± 7.6 | 0.43 |
| Serving spoons of vegetables per | 11.9 ± 5.7 | 12.5 ± 6.4 | 10.5 ± 4.7 | 9.6 ± 4.5 | 0.36 |
| week |  |  |  |  |  |
| Glasses of SSB per week | 30.0 ± 24.1 | 27.0 ± 22.2 | 21.8 ± 12.4 | 22.5 ± 14.9 | 0.27 |
|  |  |  |  |  |  |
| **Dutch Standards for dietary behavior** |  |  |  |  |  |
| Daily breakfast | 136 (82.9) | 151 (85.3) | 142 (87.1) | 151 (86.8) | 0.31 |
| Daily vegetables | 46 (28.0) | 53 (29.9) | 41 (25.5) | 34 (19.5) | 0.26 |
| Daily 3 spoons of vegetables | 13 (7.9) | 21 (11.9) | 8 (5.0) | 4 (2.3) | 0.13 |
| Daily fruit | 57 (34.8) | 68 (38.4) | 44 (27.0) | 60 (34.5) | 0.30 |
| Daily 2 pieces of fruit | 30 (18.3) | 33 (18.6) | 10 (6.1) | 9 (5.2) | 0.23 |
| Daily < 2 glasses of SSB | 37 (23.0) | 49 (28.2) | 37 (24.3) | 53 (32.3) | 0.30 |
|  |  |  |  |  |  |
| **Sedentary behavior and physical activity (mean ± SD)** |  |  |  |  |  |
| Minutes screen time per week | 707.0 ± 630.9 | 715.6 ± 536.0 | 1007.8 ± 468.2 | 1016.8 ± 416.7 | 0.27 |
| Minutes playing outside per week | 579.6 ± 424.1 | 618.1 ± 470.0 | 491.9 ± 304.1 | 445.8 ± 295.2 | 0.43 |
| Minutes physical active per week | 1056.5 ± 634.1 | 1048.2 ± 597.9 | 959.7 ± 434.9 | 882.3 ± 383.4 | 0.46 |
|  |  |  |  |  |  |
| **Dutch standards for sedentary behavior and physical activity** |  |  |  |  |  |
| Daily ≤ 120 minutes screen time | 112 (68.7) | 120 (68.6) | 57 (35.2) | 66 (37.5) | 0.17 |
| Dutch standard healthy Exercise | 143 (87.7) | 147 (83.1) | 145 (89.0) | 161 (91.5) | 0.22 |
| Play outside daily | 53 (32.5) | 60 (33.9) | 48 (29.6) | 51 (29.0) | 0.20 |
| Play outside ≥1 hour daily | 46 (28.4) | 56 (32.0) | 31 (19.0) | 30 (17.5) | 0.23 |
| Playing sport 2 days a week | 128 (78.0) | **115 (65.3)#** | 133 (81.6) | **124 (70.5)*** | 0.72 |
| Playing sports 2 days a week ≥ 30 minutes | 126 (77.3) | **110 (62.9)^** | 130 (79.8) | **120 (68.2)*** | 0.67 |
|  |  |  |  |  |  |
| **General parenting** |  |  |  |  |  |
| Authoritative parenting style | - | - | 51 (31.3) | 53 (30.1) | - |
| Authoritative parenting style (arbitrary) | - | - | 153 (93.9) | 163 (92.6) | - |
|  |  |  |  |  |  |
| **Monitoring (mean ± SD)**  **Parental feeding style score** |  |  |  |  |  |
| Control over eating | 3.6 ± 0.62 | 3.7 ± 0.70 | 4.2 ± 0.49 | 4.1 ± 0.41 | 0.23 |
| Emotional feeding | 1.6 ± 0.67 | 1.5 ± 0.68 | 1.3 ± 0.41 | 1.4 ± 0.45 | 0.17 |
| Encouragement to eat | 2.8 ± 0.81 | 2.7 ± 0.81 | 3.7 ± 0.58 | 3.6 ± 0.58 | 0.18 |
| Instrumental feeding | 1.7 ± 0.74 | 1.6 ± 0.68 | 1.5 ± 0.54 | 1.5 ± 0.58 | 0.17 |
|  |  |  |  |  |  |
| Physical activity score | - | - | 3.6 ± 0.62 | 3.6 ± 0.56 | - |
|  |  |  |  |  |  |
| **Modeling (mean ± SD)** |  |  |  |  |  |
| Intake of food score | - | - | 3.9 ± 0.42 | 4.0 ± 0.40 | - |
| Sedentary behavior score | - | - | 2.6 ± 0.50 | 2.6 ± 0.57 | - |
| Physical activity score | - | - | 3.5 ± 0.46 | 3.4 ± 0.45 | - |
|  |  |  |  |  |  |
| **Setting of rules** |  |  |  |  |  |
| Breakfast |  |  |  |  | 0.14 |
| *No* | 27 (16.6) | 28 (15.8) | 24 (14.6) | 20 (11.4) |  |
| *Yes, indulgent* | 16 (9.8) | 12 (6.8) | 4 (2.4) | 12 (6.9) |  |
| *Yes, strict* | 120 (73.6) | 137 (77.4) | 136 (82.9) | 143 (81.7) |  |
| Snacks |  |  |  |  | 0.050 |
| *No* | 37 (22.6) | 37 (20.9) | 27 (16.6) | 30 (17.1) |  |
| *Yes, indulgent* | 50 (30.5) | 56 (31.6) | 66 (40.5) | 70 (40.0) |  |
| *Yes, strict* | 77 (47.0) | 84 (47.5) | 70 (42.9) | 75 (42.9) |  |
| Vegetables |  |  |  |  | 0.12 |
| *No* | 34 (21.0) | 39 (22.0) | 23 (14.1) | 18 (10.3) |  |
| *Yes, indulgent* | 30 (18.5) | 26 (14.7) | 42 (25.8) | 56 (32.2) |  |
| *Yes, strict* | 98 (60.5) | 112 (63.3) | 98 (60.1) | 100 (57.5) |  |
| Fruit |  |  |  |  | 0.14 |
| *No* | 63 (38.7) | 78 (44.6) | 43 (26.2) | 48 (27.6) |  |
| *Yes, indulgent* | 43 (26.4) | 39 (22.3) | 48 (29.3) | 53 (30.5) |  |
| *Yes, strict* | 57 (35.0) | 58 (33.1) | 73 (44.5) | 73 (42.0) |  |
| SSBs |  |  |  |  | 0.18 |
| *No* | 56 (34.1) | 62 (35.2) | 38 (23.3) | 36 (20.6) |  |
| *Yes, indulgent* | 58 (35.4) | 43 (24.4) | 44 (27.0) | 55 (31.4) |  |
| *Yes, strict* | 50 (30.5) | 71 (40.3) | 81 (49.7) | 84 (48.0) |  |
| Watching television |  |  |  |  | 0.17 |
| *No* | 67 (41.6) | 84 (47.5) | 51 (31.3) | 60 (34.3) |  |
| *Yes, indulgent* | 52 (32.3) | 52 (29.4) | 74 (45.4) | 90 (51.4) |  |
| *Yes, strict* | 42 (26.1) | 41 (23.2) | 38 (23.3) | 25 (14.3) |  |
| Using the computer |  |  |  |  | 0.19 |
| *No* | 62 (37.8) | 57 (32.2) | 45 (27.8) | 41 (23.4) |  |
| *Yes, indulgent* | 41 (25.0) | 51 (28.8) | 69 (42.6) | 93 (53.1) |  |
| *Yes, strict* | 61 (37.2) | 69 (39.0) | 48 (29.6) | 41 (23.4) |  |
| Playing outside |  |  |  |  | -0.028 |
| *No* | 129 (78.7) | 128 (72.3) | 100 (66.3) | 117 (66.9) |  |
| *Yes, indulgent* | 23 (14.0) | 31 (17.5) | 50 (30.7) | 51 (29.1) |  |
| *Yes, strict* | 12 (7.3) | 18 (10.2) | 5 (3.1) | 7 (4.0) |  |
|  |  |  |  |  |  |
| **Parental selfefficacy (mean ± SD)** |  |  |  |  |  |
| Parenting Sense of Competence | - | - | 77.0 ± 8.5 | 76.8 ± 8.6 | - |

Unless indicated otherwise, data are presented as N (%).

* p≤0.05; # p≤0.01, ^ p≤0.001
