# Supplementary material for: The effectiveness of a web-based Dutch parenting program to prevent overweight in children 9–13 years of age: Results of a two-armed cluster randomized controlled trial
Source: PLoS One. 2022 Oct 21;17(10):e0276168. doi: 10.1371/journal.pone.0276168 (PMC9586369; doi:10.1371/journal.pone.0276168)
Supplement: S4 Appendix — (DOCX) [file pone.0276168.s004.docx]

**S4. Appendix. Effects of the e-learning on the EBRBs of the child and parenting dimensions (Completers-only)**

**Table 3.** Estimated marginal means per condition and measurement point and time by condition interactions for continuous outcomes (completers-only)

|  |  | **According to children** | | | | **According to parents** | | | |
| --- | --- | --- | --- | --- | --- | --- | --- | --- | --- |
|  |  | T0 | T1 | T2 | Time x condition | T0 | T1 | T2 | Time x condition |

|  | Condition | M (SE) | M (SE) | M (SE) | p-value | Adj. p-value | M (SE) | M (SE) | M (SE) | p-value | Adj. p-value |
| --- | --- | --- | --- | --- | --- | --- | --- | --- | --- | --- | --- |
| **Dietary behavior (days/week)** |  |  |  |  |  |  |  |  |  |  |  |
| Breakfast | CON | 6.67 (0.15) | 6.83 (0.15) | 6.66 (0.15) | 0.794 | 1.000 | 6.80 (0.05) | 6.77 (0.05) | 6.79 (0.06) | 0.526 | 1.000 |
|  | INT | 6.68 (0.15) | 6.72 (0.15) | 6.68 (0.15) |  |  | 6.79 (0.06) | 6.87 (0.06) | 6.83 (0.06) |  |  |
| Vegetables | CON | 5.71 (0.10) | 5.80 (0.10) | 5.75 (0.10) | 0.700 | 1.000 | 5.52 (0.09) | 5.43 (0.09) | 5.57 (0.09) | 0.697 | 1.000 |
|  | INT | 5.54 (0.10) | 5.77 (0.10) | 5.63 (0.10) |  |  | 5.67 (0.10) | 5.75 (0.10) | 5.76 (0.10) |  |  |
| Fruit | CON | 5.31 (0.24) | 5.28 (0.24) | 4.96 (0.24) | 0.233 | 1.000 | 5.25 (0.14) | 5.25 (0.13) | 5.08 (0.13) | 0.407 | 1.000 |
|  | INT | 5.21 (0.23) | 5.39 (0.23) | 5.05 (0.23) |  |  | 5.36 (0.14) | 5.50 (0.14) | 5.33 (0.14) |  |  |
| **Dietary behavior (amount/week)** |  |  |  |  |  |  |  |  |  |  |  |
| Serving spoons of | CON | 12.5 (0.44) | 11.7 (0.44) | 11.3 (0.44) | 0.164 | 1.000 | 9.58 (0.37) | 9.70 (0.37) | 10.2 (0.37) | 0.921 | 1.000 |
| vegetables | INT | 11.9 (0.47) | 11.9 (0.45) | 11.5 (0.47) |  |  | 10.5 (0.39) | 10.6 (0.39) | 11.0 (0.39) |  |  |
| Fruit portions | CON | 14.7 (0.73) | 15.0 (0.77) | 13.5 (0.67) | 0.575 | 1.000 | 11.4 (0.60) | 14.6 (0.60) | 14.0 (0.60) | 0.069 | 1.000 |
|  | INT | 15.0 (0.77) | 15.6 (0.73) | 14.3 (0.71) |  |  | 11.3 (0.63) | 15.5 (0.63) | 15.1 (0.63) |  |  |
| Glasses of SSBs | CON | 14.7 (0.73) | 15.0 (0.68) | 13.5 (0.67) | 0.112 | 1.000 | 22.7 (1.07) | 21.2 (1.07) | 19.9 (1.09) | 0.124 | 1.000 |
|  | INT | 15.0 (0.77) | 15.6 (0.73) | 14.3 (0.71) |  |  | 22.1 (1.13) | 19.1 (1.13) | 21.3 (1.120 |  |  |
| **Sedentary behavior** |  |  |  |  |  |  |  |  |  |  |  |
| Screen time | CON | 718 (70.5) | 660 (69.0) | 794 (75.3) | 0.606 | 1.000 | 995 (34.6) | 857 (34.8) | 1005 (34.9) | 0.846 | 1.000 |
| (min/per week) | INT | 677 (67.8) | 667 (66.7) | 718 (72.6) |  |  | 981 (36.7) | 913 (36.9) | 980 (36.5) |  |  |
| **Physical activity** |  |  |  |  |  |  |  |  |  |  |  |
| Playing outside | CON | 631 (45.1) | 622 (45.1) | 421 (45.0) | 0.031 | 0.403 | 458 (25.9) | 559 (26.0) | 378 (26.2) | 0.297 | 1.000 |
| (min/week) | INT | 599 (44.4) | 711 (44.3) | 495 (44.3) |  |  | 510 (27.5) | 614 (27.6) | 393 (27.5) |  |  |
| Physical active | CON | 1042 (90.8) | 1057 (82.0) | 977 (81.1) | 0.480 | 1.000 | 886 (32.8) | 1012 (32.9) | 896 (33.0) | 0.026 | 0.520 |
| (min/week) | INT | 1068 (87.7) | 1219 (79.5) | 1050 (78.3) |  |  | 972 (34.8) | 1063 (34.9) | 880 (34.7) |  |  |
| **General parenting** |  |  |  |  |  |  |  |  |  |  |  |
| Involvement | CON | - | - | - | - | - | 11.9 (0.26) | 12.3 (0.26) | 12.3 (0.26) | 0.084 | 1.000 |
|  | INT | - | - | - | - | - | 12.6 (0.28) | 12.4 (0.28) | 12.4 (0.28) |  |  |
| Strictness | CON | - | - | - | - | - | 7.13 (0.30) | 6.76 (0.30) | 6.56 (0.30) | 0.574 | 1.000 |
|  | INT | - | - | - | - | - | 7.15 (0.32) | 6.73 (0.32) | 6.83 (0.32) |  |  |
| **Parental feeding style** |  |  |  |  |  |  |  |  |  |  |  |
| Control over | CON | 3.68 (0.05) | 3.76 (0.05) | 3.74 (0.05) | 0.213 | 1.000 | 4.14 (0.04) | 4.10 (0.04) | 4.03 (0.04) | 0.978 | 1.000 |
| eating | INT | 3.64 (0.06) | 3.64 (0.05) | 3.63 (0.05) |  |  | 4.17 (0.04) | 4.13 (0.04) | 4.06 (0.04) |  |  |
| Emotional feeding | CON | 1.49 (0.05) | 1.44 (0.05) | 1.40 (0.05) | 0.006 | 0.084 | 1.36 (0.04) | 1.37 (0.04) | 1.30 (0.04) | 0.143 | 1.000 |
|  | INT | 1.61 (0.05) | 1.38 (0.05) | 1.32 (0.05) |  |  | 1.28 (0.04) | 1.29 (0.04) | 1.29 (0.04) |  |  |
| Encouragement | CON | 2.82 (0.06) | 2.86 (0.06) | 2.88 (0.07) | 0.340 | 1.000 | 3.63 (0.05) | 3.67 (0.05) | 3.62 (0.05) | 0.926 | 1.000 |
| to eat | INT | 2.89 (0.07) | 2.78 (0.07) | 2.86 (0.07) |  |  | 3.76 (0.05) | 3.81 (0.05) | 3.76 (0.05) |  |  |
| Instrumental | CON | 1.64 (0.05) | 1.50 (0.05) | 1.47 (0.05) | 0.340 | 1.000 | 1.51 (0.04) | 1.54 (0.04) | 1.42 (0.04) | 0.553 | 1.000 |
| feeding | INT | 1.74 (0.06) | 1.52 (0.05) | 1.42 (0.05) |  |  | 1.51 (0.05) | 1.44 (0.05) | 1.38 (0.05) |  |  |
| Monitoring | CON | 3.65 (0.05) | 3.60 (0.05) | 3.50 (0.05) | 0.171 | 0.793 | 3.65 (0.05) | 3.60 (0.05) | 3.50 (0.05) | 0.171 | 1.000 |
| physical activity | INT | 3.64 (0.05) | 3.70 (0.05) | 3.57 (0.05) |  |  | 3.64 (0.05) | 3.70 (0.05) | 3.57 (0.05) |  |  |
| **Modeling** |  |  |  |  |  |  |  |  |  |  |  |
| Intake of food | CON | - | - | - | - | - | 3.95 (0.03) | 3.93 (0.03) | 3.96 (0.03) | 0.275 | 1.000 |
|  | INT | - | - | - | - | - | 3.92 (0.03) | 3.91 (0.03) | 3.97 (0.03) |  |  |
| Sedentary | CON | - | - | - | - | - | 2.62 (0.04) | 2.68 (0.04) | 2.65 (0.04) | 0.537 | 1.000 |
| behavior | INT | - | - | - | - | - | 2.61 (0.05) | 2.67 (0.05) | 2.68 (0.05) |  |  |
| Physical activity | CON | - | - | - | - | - | 3.43 (0.04) | 3.48 (0.04) | 3.44 (0.04) | 0.808 | 1.000 |
|  | INT | - | - | - | - | - | 3.46 (0.04) | 3.51 (0.04) | 3.47 (0.04) |  |  |
| **Parental self-efficacy** |  |  |  |  |  |  |  |  |  |  |  |
| Parenting sense | CON | - | - | - | - | - | 77.0 (0.70) | 76.7 (0.69) | 78.0 (0.70) | 0.728 | 1.000 |
| of competence | INT | - | - | - |  |  | 77.1 (0.74) | 76.9 (0.74) | 77.8 (0.74) |  |  |

CON= Control condition; INT= Intervention condition

**Tabel 4** Observed probabilities per group and measurement point and time by group interactions for dichotomized outcomes (Completers-only)

|  |  | **According to children** | | | | | **According to parents** | | | | |
| --- | --- | --- | --- | --- | --- | --- | --- | --- | --- | --- | --- |
|  |  | T0 | T1 | T2 | Time x condition | | T0 | T1 | T2 | Time x condition | |
|  | Condition | Prob (SD) | Prob (SD) | Prob (SD) | p-value | Adj. p-value | Prob (SD) | Prob (SD) | Prob (SD) | p-value | Adj. p-value |
| **Dietary behavior (daily)** |  |  |  |  |  |  |  |  |  |  |  |
| Breakfast | CON | 0.85 (0.02) | 0.91 (0.02) | 0.86 (0.02) | 0.765 | 1.000 | 0.87 (0.02) | 0.86 (0.02) | 0.89 (0.02) | 0.562 | 1.000 |
|  | INT | 0.83 (0.02) | 0.87 (0.02) | 0.85 (0.02) |  |  | 0.87 (0.02) | 0.90 (0.02) | 0.91 (0.02) |  |  |
| Vegetables | CON | 0.31 (0.03) | 0.32 (0.03) | 0.32 (0.03) | 0.958 | 1.000 | 0.20 (0.02) | 0.19 (0.02) | 0.26 (0.02) | 0.721 | 1.000 |
|  | INT | 0.28 (0.02) | 0.33 (0.03) | 0.30 (0.03) |  |  | 0.25 (0.02) | 0.30 (0.03) | 0.33 (0.03) |  |  |
| > 2 portions of | CON | 0.18 (0.02) | 0.14 (0.02) | 0.15 (0.02) | 0.824 | 1.000 | 0.05 (0.01) | 0.12 (0.02) | 0.09 (0.02) | 0.302 | 1.000 |
| fruit | INT | 0.18 (0.02) | 0.19 (0.02) | 0.14 (0.02) |  |  | 0.06 (0.01) | 0.11 (0.02) | 0.12 (0.02) |  |  |
| <2 glasses of SSBs | CON | 0.28 (0.02) | 0.26 (0.02) | 0.30 (0.03) | 0.390 | 1.000 | 0.33 (0.03) | 0.30 (0.03) | 0.31 (0.03) | 0.272 | 1.000 |
|  | INT | 0.23 (0.02) | 0.24 (0.02) | 0.30 (0.03) |  |  | 0.24 (0.02) | 0.31 (0.03) | 0.29 (0.03) |  |  |
| **Sedentary behavior** |  |  |  |  |  |  |  |  |  |  |  |
| Daily ≤ 120 min | CON | 0.68 (0.03) | 0.72 (0.02) | 0.63 (0.03) | 0.618 | 1.000 | 0.37 (0.03) | 0.48 (0.03) | 0.34 (0.03) | 0.587 | 1.000 |
| screen time | INT | 0.69 (0.03) | 0.69 (0.03) | 0.67 (0.03) |  |  | 0.35 (0.03) | 0.43 (0.04) | 0.35 (0.03) |  |  |
| **Physical activity** |  |  |  |  |  |  |  |  |  |  |  |
| Dutch standard | CON | 0.63 (0.03) | 0.71 (0.03) | 0.61 (0.03) | 0.305 | 1.000 | 0.68 (0.03) | 0.71 (0.03) | 0.70 (0.03) | 0.040 | 0.880 |
| exercise | INT | 0.77 (0.02) | 0.82 (0.02) | 0.74 (0.02) |  |  | 0.80 (0.02) | 0.81 (0.02) | 0.76 (0.02) |  |  |
| Play outside >1 | CON | 0.32 (0.03) | 0.35 (0.03) | 0.17 (0.02) | 0.341 | 1.000 | 0.23 (0.02) | 0.33 (0.03) | 0.18 (0.02) | 0.927 | 1.000 |
| hour daily | INT | 0.28 (0.02) | 0.40 (0.03) | 0.18 (0.02) |  |  | 0.25 (0.02) | 0.42 (0.34) | 0.19 (0.02) |  |  |
| **General parenting** |  |  |  |  |  |  |  |  |  |  |  |
| Authoritative | CON | - | - | - | - |  | 0.30 (0.03) | 0.31 (0.03) | 0.31 (0.03) | 0.821 | 1.000 |
| parenting style | INT | - | - | - |  |  | 0.31 (0.03) | 0.28 (0.02) | 0.31 (0.03) |  |  |
| **Setting of rules** |  |  |  |  |  |  |  |  |  |  |  |
| Breakfast |  |  |  |  |  |  |  |  |  |  |  |
| No | CON | 0.17 (0.02) | 0.19 (0.03) | 0.14 (0.02) | 0.336 | 1.000 | 0.10 (0.02) | 0.07 (0.02) | 0.10 (0.02) | 0.688 | 1.000 |
|  | INT | 0.16 (0.02) | 0.21 (0.03) | 0.19 (0.03) |  |  | 0.11 (0.02) | 0.14 (0.03) | 0.10 (0.02) |  |  |
| Indulgent | CON | 0.06 (0.02) | 0.08 (0.02 | 0.11 (0.02) |  |  | 0.08 (0.02) | 0.07 (0.02) | 0.11 (0.02) |  |  |
|  | INT | 0.11 (0.02) | 0.13 (0.02) | 0.12 (0.02) |  |  | 0.07 (0.02) | 0.05 (0.02) | 0.07 (0.02) |  |  |
| Strict | CON | 0.77 (0.03) | 0.74 (0.03) | 0.74 (0.03) | 0.607 | 1.000 | 0.82 (0.03) | 0.86 (0.03) | 0.79 (0.03) | 0.846 | 1.000 |
|  | INT | 0.73 (0.03) | 0.66 (0.03) | 0.69 (0.03) |  |  | 0.83 (0.02) | 0.81 (0.03) | 0.84 (0.03) |  |  |
| Snacks |  |  |  |  |  |  |  |  |  |  |  |
| No | CON | 0.24 (0.03) | 0.19 (0.03) | 0.22 (0.03) | 0.753 | 1.000 | 0.16 (0.03) | 0.11 (0.02) | 0.14 (0.02) | 0.621 | 1.000 |
|  | INT | 0.21 (0.03) | 0.20 (0.03) | 0.26 (0.03) |  |  | 0.16 (0.03) | 0.16 (0.03) | 0.14 (0.02) |  |  |
| Indulgent | CON | 0.31 (0.03) | 0.40 (0.03) | 0.35 (0.03) |  |  | 0.40 (0.03) | 0.43 (0.04) | 0.44 (0.04) |  |  |
|  | INT | 0.32 (0.03) | 0.39 (0.03) | 0.36 (0.03) |  |  | 0.43 (0.03) | 0.47 (0.04) | 0.45(0.04) |  |  |
| Strict | CON | 0.45 (0.03) | 0.40 (0.03) | 0.43 (0.03) | 0.341 | 1.000 | 0.43 (0.03) | 0.45 (0.04) | 0.42 (0.04) | 0.796 | 1.000 |
|  | INT | 0.48 (0.03) | 0.41 (0.03) | 0.38 (0.03) |  |  | 0.41 (0.03) | 0.37 (0.04) | 0.41 (0.03) |  |  |
| Vegetables |  |  |  |  |  |  |  |  |  |  |  |
| No | CON | 0.21 (0.03) | 0.22 (0.03) | 0.21 (0.03) | 0.230 | 1.000 | 0.11 (0.02) | 0.08 (0.02) | 0.13 (0.02) | 0.491 | 1.000 |
|  | INT | 0.20 (0.03) | 0.29 (0.03) | 0.27 (0.03) |  |  | 0.13 (0.02) | 0.15 (0.03) | 0.11 (0.02) |  |  |
| Indulgent | CON | 0.17 (0.03) | 0.20 (0.03) | 0.21 (0.03) |  |  | 0.29 (0.03) | 0.31 (0.03) | 0.28 (0.03) |  |  |
|  | INT | 0.20 (0.03) | 0.24 (0.03) | 0.24 (0.03) |  |  | 0.27 (0.03) | 0.28 (0.03) | 0.23 (0.03) |  |  |
| Strict | CON | 0.62 (0.03) | 0.59 (0.03) | 0.59 (0.03) | 0.149 | 1.000 | 0.60 (0.03) | 0.61 (0.04) | 0.59 (0.04) | 0.378 | 1.000 |
|  | INT | 0.59 (0.03) | 0.48 (0.03) | 0.49 (0.03) |  |  | 0.59 (0.03) | 0.57 (0.04) | 0.66 (0.03) |  |  |
| Fruit |  |  |  |  |  |  |  |  |  |  |  |
| No | CON | 0.44 (0.03) | 0.43 (0.03) | 0.45 (0.03) | 0.149 | 1.000 | 0.30 (0.03) | 0.22 (0.03) | 0.28 (0.03) | 0.416 | 1.000 |
|  | INT | 0.38 (0.03) | 0.45 (0.03) | 0.49 (0.03) |  |  | 0.24 (0.03) | 0.23 (0.03) | 0.21 (0.03) |  |  |
| Indulgent | CON | 0.21 (0.03) | 0.24 (0.03) | 0.22 (0.03) |  |  | 0.28 (0.03) | 0.37 (0.04) | 0.33 (0.03) |  |  |
|  | INT | 0.26 (0.03) | 0.26 (0.03) | 0.24 (0.03) |  |  | 0.34 (0.03) | 0.31 (0.04) | 0.35 (0.03) |  |  |
| Strict | CON | 0.35 (0.03) | 0.33 (0.03) | 0.32 (0.03) | 0.303 | 1.000 | 0.42 (0.03) | 0.41 (0.04) | 0.39 (0.04) | 0.390 | 1.000 |
|  | INT | 0.36 (0.03) | 0.29 (0.03) | 0.27 (0.03) |  |  | 0.42 (0.03) | 0.46 (0.04) | 0.44 (0.03) |  |  |
| SSBs |  |  |  |  |  |  |  |  |  |  |  |
| No | CON | 0.37 (0.03) | 0.35 (0.03) | 0.36 (0.03) | 0.576 | 1.000 | 0.21 (0.03) | 0.18 (0.03) | 0.14 (0.03) | 0.713 | 1.000 |
|  | INT | 0.33 (0.03) | 0.29 (0.03) | 0.32 (0.03) |  |  | 0.21 (0.03) | 0.19 (0.03) | 0.18 (0.03) |  |  |
| Indulgent | CON | 0.24(0.03) | 0.22 (0.03) | 0.31 (0.03) |  |  | 0.30 (0.03) | 0.30 (0.03) | 0.41 (0.04) |  |  |
|  | INT | 0.33 (0.03) | 0.36 (0.03) | 0.35 (0.03) |  |  | 0.30 (0.03) | 0.28 (0.03) | 0.38 (0.03) |  |  |
| Strict | CON | 0.39 (0.03) | 0.44 (0.03) | 0.33 (0.03) | 0.111 | 1.000 | 0.49 (0.03) | 0.51 (0.04) | 0.45 (0.04) | 0.506 | 1.000 |
|  | INT | 0.34(0.03) | 0.35 (0.03) | 0.32 (0.03) |  |  | 0.49 (0.03) | 0.53 (0.04) | 0.44 (0.04) |  |  |
| Watching television |  |  |  |  |  |  |  |  |  |  |  |
| No | CON | 0.48 (0.03) | 0.49 (0.03) | 0.48 (0.03) | 0.840 | 1.000 | 0.36 (0.03) | 0.30 (0.03) | 0.35 (0.03) | 0.093 | 1.000 |
|  | INT | 0.45 (0.03) | 0.47 0.03) | 0.43 (0.03) |  |  | 0.34 (0.03) | 0.23 (0.03) | 0.25 (0.03) |  |  |
| Indulgent | CON | 0.29 (0.03) | 0.26 (0.03) | 0.35 (0.03) |  |  | 0.48 (0.03) | 0.54 (0.04) | 0.48 (0.04) |  |  |
|  | INT | 0.34 (0.03) | 0.28 (0.03) | 0.35 (0.03) |  |  | 0.44 (0.03) | 0.53 (0.04) | 0.50 (0.04) |  |  |
| Strict | CON | 0.23 (0.03) | 0.25 (0.03) | 0.17 (0.03) | 0.640 | 1.000 | 0.16 (0.02) | 0.16 (0.03) | 0.17 (0.03) | 0.697 | 1.000 |
|  | INT | 0.22 (0.03) | 0.25 (0.03) | 0.22 (0.03) |  |  | 0.22 (0.03) | 0.23 (0.03) | 0.24(0.03) |  |  |
| Using the computer |  |  |  |  |  |  |  |  |  |  |  |
| No | CON | 0.35 (0.03) | 0.34 (0.03) | 0.41 (0.03) | 0.100 | 1.000 | 0.26 (0.03) | 0.24 (0.03) | 0.26 (0.03) | 0.029 | 0.667 |
|  | INT | 0.39 (0.03) | 0.37 (0.03) | 0.40 (0.03) |  |  | 0.28 (0.03) | 0.19 (0.03) | 0.22 (0.03) |  |  |
| Indulgent | CON | 0.28 (0.03) | 0.36 (0.03) | 0.32 (0.03) |  |  | 0.48 (0.03) | 0.50 (0.04) | 0.50 (0.04) |  |  |
|  | INT | 0.25 (0.03) | 0.29 (0.03) | 0.30 (0.03) |  |  | 0.46 (0.03) | 0.45 (0.04) | 0.44 (0.04) |  |  |
| Strict | CON | 0.37 (0.03) | 0.30 (0.03) | 0.27 (0.03) | 0.100 | 1.000 | 0.25 (0.03) | 0.26 (0.03) | 0.24 (0.03) | 0.362 | 1.000 |
|  | INT | 0.35 (0.03) | 0.34 (0.03) | 0.31 (0.03) |  |  | 0.26 (0.03) | 0.35 (0.04) | 0.34 (0.03) |  |  |
| Playing outside |  |  |  |  |  |  |  |  |  |  |  |
| No | CON | 0.73 (0.03) | 0.75 (0.03) | 0.77 (0.03) | 0.505 | 1.000 | 0.64 (0.03) | 0.54 (0.04) | 0.63 (0.03) | 0.147 | 1.000 |
|  | INT | 0.77 (0.03) | 0.83 (0.03) | 0.80 (0.03) |  |  | 0.63 (0.03) | 0.50 (0.04) | 0.56 (0.04) |  |  |
| Indulgent | CON | 0.16 (0.02) | 0.14 (0.02) | 0.14 (0.02) |  |  | 0.31 (0.03) | 0.42 (0.04) | 0.32 (0.03) |  |  |
|  | INT | 0.14 (0.02) | 0.12(0.02) | 0.14(0.02) |  |  | 0.32 (0.03) | 0.39 (0.04) | 0.36 (0.03) |  |  |
| Strict | CON | 0.11 (0.02) | 0.11 (0.02) | 0.09 (0.02) | 0.955 | 1.000 | 0.05(0.01) | 0.04 (0.02) | 0.05 (0.02) | 0.010 | 0.240 |
|  | INT | 0.10 (0.02) | 0.06 (0.02) | 0.07 (0.02) |  |  | 0.05 (0.01) | 0.10 (0.02) | 0.09 (0.02) |  |  |

The multicategory variables for parental EBRB rules were dichotomized in order to compare between “no rules” and “indulgent/strict rules” and between “strict rules” and “no/indulgent rules”. Therefore 2 p-values for each parental EBRB rules are presented.
