# Supplementary material for: The effectiveness of a web-based Dutch parenting program to prevent overweight in children 9–13 years of age: Results of a two-armed cluster randomized controlled trial
Source: PLoS One. 2022 Oct 21;17(10):e0276168. doi: 10.1371/journal.pone.0276168 (PMC9586369; doi:10.1371/journal.pone.0276168)
Supplement: S5 Appendix — (DOCX) [file pone.0276168.s005.docx]

**S5 Appendix. Process evaluation**

**
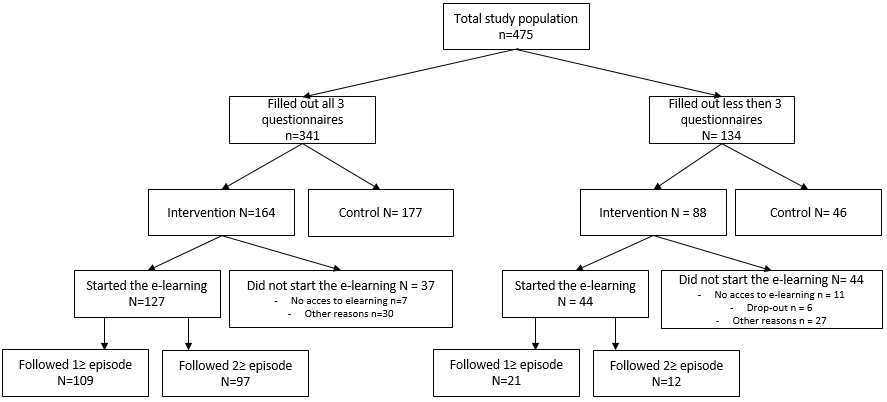
**

**Fig 2. Flowchart following the e-learning by parent-child dyads.**

**Table 5. Following the e-learning by parent-child dyads**

|  |  | **Condition** | | **Access to e-learning** | | **Started the e-learning** | | **Number of episodes followed** | | |
| --- | --- | --- | --- | --- | --- | --- | --- | --- | --- | --- |
| **Framework study population** | **Total**  **n** | **Intervention**  **n** | **Control**  **n** | **Yes**  **N (%)** | **No**  **N (%)** | **Yes**  **N (%)** | **No**  **N (%)** | **1≥ episode**  **N (%)** | **2≥ episodes**  **N (%)** | **All 5 episodes**  **N (%)** |
| **Intention-to-treat** | 475 | 252 | 223 | 234 (92.9) | 18^a^ (7.1) | 171 (67.9) | 81 (32.1) | 130 (51.6) | 109 (43.3) | 85 (33.7%) |
| **Completers-only** | 341 | 164 | 177 | 157 (95.7) | 7^b^ (4.3) | 127 (77.4) | 37 (22.6) | 109 (66.5) | 97 (59.1) | 72 (43.9%) |

All percentages were calculated using total parent-child dyads in the intervention condition, ^a^ n=6 decline to participate, n= 18 incorrect e-mail address; ^b^ n=7 incorrect e-mail address.

**Table 6. Parent’s willingness to follow the e-learning**

|  | **Did not start the e-learning**  **N (%)** | **Did start the e-learning**  **N (%)** | **Chi-square**  **p-value** | **Followed 2 or more episodes**  **N (%)** | **Followed less than 2 episodes**  **N (%)** | **Chi-square**  **p-value** |
| --- | --- | --- | --- | --- | --- | --- |
| **Number of parent-child dyads** | 81 (32.1) | 171 (67.8) |  | 109 (43.3) | 143 (56.7) |  |
| **Age of the child (years)** |  |  |  |  |  |  |
| 8 years | 1 (33.3) | 2 (66.7) |  | 1 (33.3) | 2 (66.7) |  |
| 9 years | 18 (25.4) | 53 (74.6) |  | 33 (46.5) | 38 (53.5) |  |
| 10 years | 15 (31.9) | 32 (68.1) |  | 23 (48.9) | 24 (51.1) |  |
| 11 years | 30 (33.3) | 60 (66.7) |  | 41 (45.6) | 49 (54.4) |  |
| 12 years | 13 (37.1) | 22 (62.9) |  | 11 (31.4) | 24 68.6) |  |
| 13 years | 4 (66.7) | 2 (33.3) | 0.387 | 0 (0.0) | 6 (100) | 0.168 |
| **Gender of the child** |  |  |  |  |  |  |
| Male | 39 (28.7) | 97 (71.3) |  | 60 (44.1) | 76 (55.9) |  |
| Female | 42 (36.2) | 74 (63.8) | 0.202 | 49 (42.2) | 67 (57.8) | 0.764 |
| **Ethnicity** |  |  |  |  |  |  |
| Caucasian | 74 (30.6) | 168 (69.4) |  | 108 (44.6) | 134 (55.4) |  |
| Non-Caucasian | 7 (70) | 3 (30) | **0.009** | 1 (10) | 9 (90) | **0.030** |
| **Level of education of parents** |  |  |  |  |  |  |
| Low | 30 (38.0) | 49 (62.0) |  | 28 (35.4) | 51 (64.6) |  |
| Middle | 43 (33.9) | 84 (66.1) |  | 56 (44.1) | 71 (55.9) |  |
| High | 7 (15.6) | 38 (84.4) | **0.029** | 25 (55.6) | 20 (44.4) | 0.092 |
| **Weight status of the child** |  |  |  |  |  |  |
| Overweight/obese | 14 (31.8) | 30 (68.2) |  | 22 (50) | 22 (50) |  |
| No overweight | 65 (32.0) | 138 (68.0) | 0.979 | 85 (41.9) | 118 (58.1) | 0.324 |
| **Weight status of the parent** |  |  |  |  |  |  |
| Overweight/obese | 33 (33.9) | 72 (66.1) |  | 42 (38.5) | 67 (61.5) |  |
| No overweight | 37 (28.7) | 92 (71.3) | 0.382 | 63 (48.) | 66 (51.2) | 0.111 |
| **Perception of the weight status of the child** |  |  |  |  |  |  |
| (Extremely) low | 7 (29.2) | 17 (70.8) |  | 10 (41.7) | 14 (58.3) |  |
| Normal | 65 (32.8) | 133 (67.2) |  | 84 (42.2) | 114 (57.5) |  |
| (Extremely) heavy | 8 (29.6) | 19 (70.4) | 0.884 | 14 (51.9) | 13 (48.1) | 0.487 |
