# Supplementary material for: The effectiveness of a web-based Dutch parenting program to prevent overweight in children 9–13 years of age: Results of a two-armed cluster randomized controlled trial
Source: PLoS One. 2022 Oct 21;17(10):e0276168. doi: 10.1371/journal.pone.0276168 (PMC9586369; doi:10.1371/journal.pone.0276168)
Supplement: Attachment — Submitted filename: 20220204 Response to editor and reviewer.docx [file pone.0276168.s006.docx]

**Response to Editor and Statistical reviewer**

PONE-D-21-30893
The effectiveness of a web-based Dutch parenting program to prevent overweight in children 9-13 years of age: results of a two-armed cluster randomized controlled trial

**Response to comments Academic Editor PLOS ONE: Lee Van Horn, PhD**

Dear Dr. Ruiter,

Thank you for submitting your manuscript to PLOS ONE. After careful consideration, we feel that it has merit but does not fully meet PLOS ONE’s publication criteria as it currently stands. Therefore, we invite you to submit a revised version of the manuscript that addresses the points raised during the review process.

The decision for this paper is a difficult one. Let me start by saying that I believe it is important to publish this paper, its just a difficult situation. On the one hand you have taken the appropriate steps, registering the clinical trial, publishing a study protocol, and then for the most part following that protocol in this paper. On the other hand, I have many of the same concerns that are raised by the statistical reviewer as well as a few others. I think you should know where I’m coming from as an editor, while I’m trained as a social scientist, my primary role is as a methodologist, I’ve been the primary statistical methods person on multiple randomized and group randomized trials, some targeting physical activity and weight loss. I am also a reviewer for major clinical trials grants in the area. If I had reviewed either the grant application or the study protocol paper I would have had reservations about the statistical methods proposed (doing analyses in Mplus with TYPE = COMPLEX, failure to account for multiple testing, dichotomizing outcomes, treating T1 and T2 as separate outcomes, reporting both ITT and completers only analysis) and would have requested major revisions. So, now the question I’m facing is how to reconcile the desire to stay true to the published protocol paper versus moving forward with results that do not meet the standard I would expect from a clinical trial. My overall decision at this point is to request major revisions which try to address these issues. I’ll also note that I held off sending this paper out to content specific reviewers because I wanted to see if the statistics reviewer had the same concerns I did, and because the substantive reviews will likely be better if these other issues are fixed.

Several things seem straight forward to me:

1) the protocol paper did not include per-protocol analyses, I believe that these need to be dropped or if they are retained they need to be done using Complier Average Causal Effects analyses appropriate for cluster randomized trials (there is a paper out there on doing this by Booil Jo), but these are difficult analyses to do and I would want the code and output included so that they could be checked;

- *Thank you for your advice. For this reason we removed the per-protocol analyses. In the Method section in line 372-374 we removed the text “, and in a per-protocol framework (i.e., parent-child dyads who completed all three questionnaires and parents in the intervention group who completed at least two of the e-learning modules)”.*
- *In the result section in line 513-525 and in line 564-574 we removed the results of the per-protocol analyses.*
- *In the discussion section in line 618-619 we removed the text “and per protocol analyzes”.*
- *In the abstract in line 41-45 and the discussion section in line 621-625 we removed the text “Per protocol analyses also revealed additional positive effects with respect to the child’s consumption of fruits and vegetables, playing outside, being physical active, and the parents’ use of an authoritative general parenting style, parental control over eating, monitoring of the child’s physical activity, and higher satisfaction regarding parental self-efficacy”.*

2) the protocol paper did not mention how multiple outcomes would be tested, this is really a major problem, you have 13 outcomes multiplied by two different reporters (parents and children) and two different time points, so 52 different analyses for 3 different types of analyses. The trial registration includes 7 primary outcomes, only a little bit better. This needs to be addressed and I’m not sure that you have many options at this point, but the end result is likely that there are no reliable differences between treatment and control groups;

- *Thank you for this comment. We reduced our reported primary outcomes to 7, as mentioned in our protocol paper.*
- *The 13 outcomes we reported in our in our first submitted main manuscript were:*
  1. *Breakfast (days/week), daily Breakfast*
  2. *Vegetables (days and amount/week), Daily vegetables*
  3. *Daily 3 serving spoons of vegetables*
  4. *Fruit (days and amount/week), Daily Fruit*
  5. *Daily 2 pieces of fruit*
  6. *SSB (days and glasses/week), Daily <2 glasses SSBs*
  7. *Television (days and minutes/week)*
  8. *Computer (days and minutes/week)*
  9. *Screentime (days and minutes/week), Daily ≤120 min screen time*
  10. *Play outside (days and minutes/week), Daily Play outside*
  11. *Daily Play outside > 1 hour*
  12. *Physical active (days and minutes/week), Dutch standard healthy exercise (participating in an organized sport for ≥30 minutes at least twice a week)*
  13. *Walk/cycle to school (days/week)*
- *In our revised manuscript we now reported the following 7 primary outcomes as mentioned in the protocol paper:*

1. *Breakfast (days/week), daily Breakfast*
2. *Vegetables (days and amount/week), Daily vegetables*
3. *Fruit (days and amount/week), Daily 2 pieces of fruit*
4. *SSB (days and glasses/week), Daily <2 glasses SSBs*
5. *Screentime (days and minutes/week), Daily ≤120 min screen time*
6. *Play outside (days and minutes/week), Daily Play outside > 1 hour*
7. *Physical active (days and minutes/week), Dutch standard healthy exercise (participating in an organized sport for ≥30 minutes at least twice a week)*

- *Furthermore we now corrected for multiple outcome. P-values of interaction effects were corrected using Holm-Bonferroni correction. The p-values for continuous outcomes reported by children are corrected for the number of p-values for continuous outcomes reported by children. The p-values for continuous outcomes reported by parents are corrected for the number of p-values for continuous outcomes reported by parents. We did the same for the dichotomous variables. Pairwise comparisons were adjusted using Tukey-method to adjust for multiple testing. Accordingly, in the abstract in line 38 we added the text “after correction for multiple testing”, in the method section in line 408-409 we added the text: “To adjust for multiple testing, the p-values of the time x condition interaction effects were adjusted using Holm-Bonferroni sequential correction.” In the method section in line 418-419 we added the text: “For pairwise comparison, p-values were adjusted using the Tukey-method to adjust for multiple testing.”*
- *In the result section in line 488 and in line 537 we added the test “, and after adjusting for multiple testing,”*
- *In the Discussion section in line 709-721 we deleted the text:* *“First, it is worth noting that we did not apply Bonferroni correction for multiple testing, as this was the first study to examine the effects of an e-learning program for parents. In this exploratory study, we examined the effects of all relevant variables for which we expected the e-learning program to have an effect. Moreover, we did not expect the program to have particularly large effects, and we did not want to prematurely conclude that the e-learning program did not have an effect. Furthermore, we anticipated wide variations with respect to the outcome measures, as the e-learning program could have had affected slightly different variables among the children and parents (for example, the consumption of SSBs but not fruit could have been affected in one child, while the amount of screen time could have been affected in another child). Thus, applying Bonferroni correction may have been overly strict, as this conservative method can obscure significant results unless the effects and/or sample sizes are extremely large. Furthermore, this approach can increase the likelihood of a type II error, rendering a bona fide difference non-significant [69].”*
- *In addition, the following secondary outcomes were measured and reported in the revised manuscript:*
  1. *parental styles*
  2. *parenting practices*
  3. *parental self-efficacy*
  4. *parents’ willingness to follow the e-learning program*
  5. *parents’ satisfaction with the e-learning program*

3) Your actual statistical analyses need to be made clear. You simply report that you used Mplus with type=complex, this is not the statistical analysis, it is just the software used. As the statistical reviewer mentions, the models used need to be reported in careful detail with all predictors and outcomes specified, assumptions tested, and variance components reported;

- *Thank you for this comment.* *Based on your comments we decided to revise our strategy for analyses and to redo our analyses. We replaced the analyses in Mplus with linear mixed models in R. We now also more clearly describe how the analyses were conducted.*
- *The Statistical analyses section has been rewritten. In the method section in line 379-389 we deleted the text about the analysis in Mplus and replaced this text* *in line 374-379 by “To determine the effectiveness of the intervention, we used linear mixed models in R [66], instead of the planned regression analyses in Mplus as described in the study protocol [40]. Linear mixed models were chosen for two reasons: i) because of the nested data structure of the data (i.e., repeated measurements nested within participants, and participants nested in schools), and ii) because multi-level analyses do not delete participants listwise, and can more appropriately handle data missing at random [67, 68].”. And in line 389-419 “**The multicategory variables for parental EBRB rules were dichotomized in order* *to compare between “no rules” and “indulgent/strict rules” and between “strict rules” and “no/indulgent rules”, providing better interpretation of data.* *Linear mixed models for continuous outcomes and generalized linear mixed models with logit links for binary outcomes were used. The package lme4 was used for both LMMs and GLMMs [69].* *All models included a random effect for students to account for repeated measurements within participants. For each outcome, it was also tested whether it is necessary to additionally account for nesting of students within schools. This additional random factor was only included if the intraclass correlation for the school-level was higher than .05, suggesting significant clustering of students within schools [70, 71]. In addition, it was tested whether a random intercept and slope model significantly better fit the data than the more parsimonious random intercept model using log-likelihood ratio tests. If this was the case, the model with random slopes was used. If the more complex models including additional random factors and/or random slopes did not converge or were singular, we used a more parsimonious model, for example by only modelling random intercepts. Time, condition and their higher-order interactions were included as fixed effects in all models. An unstructured covariance structure was used for all models. Because there were two possible starting points of the study, namely i) February 2013, in which participants of 9 schools were randomized, and ii) September 2013, in which participants of another 2 schools were randomized, the variable ‘moment of randomization’ was included as covariate to correct for possible seasonal effects. To adjust for multiple testing, the p-values of the time x condition interaction effects were adjusted using Holm-Bonferroni sequential correction. All models were visually inspected for homogeneity and normal distribution of errors using residuals versus fitted value plots and Q-Q plots. If it was suspected that these assumptions might have been violated, or if a continuous outcome was strongly left or right skewed, we additionally fitted the models with beta-regression using the glmmTMB package [68]. The results from the beta-regression models were then compared with linear mixed models. Since the outcomes from the linear beta models did not lead to substantially different conclusions, only linear mixed models were reported for continuous outcomes. To determine treatment effects at each of the follow-up timepoints, pairwise post-hoc comparisons of estimated marginal means by time and condition were performed using the emmeans package. For paiwise comparison, p-values were adjusted using the Tukey-method to account for multiple testing. An α of 0.05 was used to indicate statistical significance for all analyses.”*

4) the justification for the sample size calculations needs to be made in much more detail including both the ICC assumptions and the effect size assumptions along with references;

- *Thank you for this comment.* *We assume an intraclass correlation coefficient (ICC) of 0.05. This estimate is based on a study on ICCs for obesity indicators in primary schools (Gray et al., 2016). The ICC’s for most outcomes varied between 0.02 and 0.05. Based on this study we decided to use an ICC of 0.05 for the power analysis.*
- *As for the effect size assumption:* *The assumption of an increase of 20% is based on previous effectiveness studies and school-based programs in children 9-13 years of age [48-51]”.*
- *48. De Lijster-van Kampen GPA, Gomez-Tromp M, Kocken PL: Evaluatie Door Dik en Dun: Resultaten van een pilotonderzoek naar de effecten van een behandelprogramma bij kinderen met overgewicht of obesitas in de leeftijd van 8-12 jaar [Results of a pilot study on the effects of a treatment program for overweight or obese children aged 8-12 years]. In.: TNO Kwaliteit van Leven; 2010.*
- *49. Howerton MW, Bell BS, Dodd KW, Berrigan D, Stolzenberg-Solomon R, Nebeling L: School-based nutrition programs produced a moderate increase in fruit and vegetable consumption: meta and pooling analyses from 7 studies. J Nutr Educ Behav 2007, 39(4):186-196.*
- *50. Reynolds KD, Franklin FA, Binkley D, Raczynski JM, Harrington KF, Kirk KA, Person S: Increasing the fruit and vegetable consumption of fourth-graders: results from the high 5 project. Prev Med 2000, 30(4):309-319.*
- *51. Robinson TN: Reducing children's television viewing to prevent obesity: a randomized controlled trial. Jama 1999, 282(16):1561-1567.*
- *We refer here to the text in our protocol paper: “Sample size was calculated based on the difference between the intervention group and the control group in terms of the following outcomes: improvement in healthy eating and a reduction in sedentary behavior in children in accordance with Dutch standards. Because the e-learning program has not been tested previously with respect to effectiveness, it is difficult to estimate an effect size. However, based on the outcomes of similar effectiveness studies and school-based programs in children 9-13 years of age [16-19], we expect a minimum difference of 20% between the control and intervention groups 12 months after baseline in the children’s dietary and/or sedentary behavior in accordance with the Dutch standards.”.*
- *In the method section in line 214-215 we deleted the text: “(with an estimated intraclass correlation coefficient of 0.05 and an average cluster size of 14).”. and in line 215-217 we added the text “We assume an average cluster size of 14 and an intraclass correlation coefficient (ICC) of 0.05* *based on a study on ICCs for obesity indicators in primary schools in which the ICCs for most outcomes were between 0.02 and o.05 [47].”. And in line 221-223 we added the text “The assumption of an increase of 20% is based on previous effectiveness studies and school-based programs in children 9-13 years of age [48-51]”.*

5) Missing data and imputation needs to be described in more detail.

- *Thank you for this comment. We replaced our current analyses in Mplus with analyses with linear mixed models in R, that more appropriately handles missing data at random. We rewrote our Statistical analyses section to make this clearer. In the method section in line 379-389 we deleted the text about the analysis in Mplus and replaced this text in line 374-379 by “To determine the effectiveness of the intervention, we used linear mixed models in R [66], instead of the planned regression analyses in Mplus as described in the study protocol [40]. Linear mixed models were chosen for two reasons: i) because of the nested data structure of the data (i.e., repeated measurements nested within participants, and participants nested in schools), and ii) because multi-level analyses do not delete participants listwise, and can more appropriately handle data missing at random [67, 68].”*

Now the difficult things. First of all is your use of Mplus and type=complex to account for the clustering in the data. I’m going to start by nothing that you are wrong about this being multilevel analysis, when you use the type=complex option Mplus simply adjusts the standard errors of your effects for clustering using a sandwich estimator, but this is not a multilevel regression model. The first reason I don’t typically suggest Mplus for multilevel models is that users often don’t understand what is happening, the second is that many Mplus features are not well documented and it really isn’t clear what sandwich estimator is employed. In any case, this should be similar to more typical implementations of GEE. While GEE can be acceptable in GRTs for correcting for clustering, it isn’t very efficient and the approach recommended by the reviewer is clearly the standard. A recent paper by Turner et al (American J of Public Health) discusses some of these issues. If the protocol paper wasn’t published I would feel quite strongly (along with the stats reviewer) that the current analyses should be completely replaced with the conventional multilevel models recommended by the reviewer. However, given the existence of the protocol paper, my suggestion is that the ITT and completers analyses stays as is (with the full model including all covariates and variance parameter estimates described) but that the analyses suggested by the reviewer be included in an appendix and any discrepancies noted. There really shouldn’t be large discrepancies if everything was done right, and my reason for asking for this is that it will greatly increase my confidence that everything was done right.

- *Thank you for your comment and advice. Based on your comments we decided to revise our strategy for analyses and to use the multilevel models recommended by you and the statistical reviewer. As we reported as answer on you question 3, we have rewritten our Statistical analyses section, see in the Method section in line 374- 419 (see above), and we now report the results of these new analyses in the Results section in line 482-492 “The effects of the e-learning program on the children’s EBRBs and parenting dimensions according to the results of the intention-to-treat analysis are summarized in Tables 3 and 4, and the completers-only sensitivity analyses are shown in Additional file 4 (S4 Appendix)* ***Effects of the e-learning program on the children’s EBRBs***
- *Based on both the children’s responses and the parents’ responses to the questionnaires, and after adjusting for multiple testing, we found no significant differences in the changes over time between the intervention and control condition with respect to the children’s diet, sedentary behavior or physical activity behavior. In addition, post-hoc pairwise comparisons showed no significant difference between conditions at any of the follow-up times.” and in line 533-541 “****Effects of the e-learning program on parenting dimensions***
- *Based on both the children’s responses and the parents’ responses to the questionnaires, and after adjusting for multiple testing, we found no significant differences in the change over time between the intervention and control condition with respect to parenting dimensions, including general parenting, specific EBRB-guided parenting (e.g., monitoring, modeling, and establishing EBRB rules), or parental self-efficacy. In addition, post hoc pairwise comparisons showed no significant difference between conditions at any of the follow-up times.” and in line 577 Table 3 and Table 4 .*
- *We decided not to report our analyses in Mplus in an additional file. This decision was made because of the limitations of these analyses as pointed out by the reviewer and editor, and to make the manuscript more readable.*

Second, were covariates included in these analyses? Also, was the analyses the outcome at time T controlling for baseline or was it change scores? If covariates were not included, why not? I agree with the reviewer that baseline values for weight as other variables should have been included, the protocol paper is not clear about how or if this will be done.

- *In our Mplus analyses no covariates were included, because there were no differences at baseline between the variables.*
- *The analyses in Mplus measured the change scores.*
- *In the revised manuscript we replaced the analyses in Mplus with linear mixed models in R. Time, condition and their higher-order interactions were included as fixed effects in all models. An unstructured covariance structure was used for all models.* *Because there were two possible starting points of the study (i) participants of 9 schools who were randomized in February 2013, ii) and participant of another 2 schools who were randomized in September 2013), the variable ‘moment of randomization’ was included as covariate to correct for possible seasonal effects. And in the method section in line 403-408 we added the text: “Time, condition and their higher-order interactions were included as fixed effects in all models. An unstructured covariance structure was used for all models.* *Because there were two possible starting points of the study, namely i) February 2013, in which participants of 9 schools were randomized, and ii) September 2013, in which participants of another 2 schools were randomized, the variable ‘moment of randomization’ was included as covariate to correct for possible seasonal effects.”*

Third, I’m not sure that all of your effect sizes are even in the correct direction for the few significant treatment effects. Looks like there were more sweetened beverages consumed in the treatment than control groups? Also, some of the significance tests in the completers only table seem to be off? Oh, the use of superscripts to indicate which effects are significant for which set of analyses doesn’t work at all. You should probably just report the full results of the ITT and then, separately, of the completers-only analyses (again, dropping the protocol analyses).

- *Thank you for this comment. As mentioned above, based on your comments we decided to revise our strategy for analyses and we replace our analyses with linear mixed models that account for the multilevel structure of the data as recommended by you and the statistical reviewer.*
- *We replaced table 3-6 (with the original results of the M-plus analyses) with the results of our analyses in R. In our new tables (table 3 and 4) we show the results of our intention-to-treat analyses. In these tables we don’t use superscripts to indicate which effects were significant in the completers only analyses. The two tables with the results of our completers only analyses are placed in the additional file to make the manuscript easier to read.*
- *In the additional file S4 Appendix we report the full results of the completers-only analyses.*

I’ve noted several other issues. One is that I doubt that substantive reviewers would be impressed with your outcomes for the dietary or physical activity measures. On the trials I’ve been on we have ignored self or parent reported PA and focused exclusively on accelerometer measured PA because of concerns about bias as well as unreliability. Obviously you can’t change this now, but it needs to be a very important discussion point and the issues with the measures need to be made very clear.

- *Thank you for this comment and advice. In our main manuscript in the Discussion section in line 727-735 we mentioned this study limitation: “Fourth, the information regarding the behaviors of the children and parents was obtained entirely using self-reporting questionnaires completed by the children and parents, which may have resulting in over-reporting and/or under-reporting due social desirability and/or recall bias. To minimize social desirability and optimize measurement validity, we stressed full confidentiality (i.e., anonymity) to our participants. To minimize recall bias, the interval between the events being recalled and the questionnaire was relatively short (participants were asked to recall events from the past month or week), which likely also increased the self-reporting reliability.”*
- *Because of your advice in the Discussion section in line 730-731 we added the text “and may contribute to unreliability of the reported data [80].”, and in line 735-737 we added the text “ To increase reliability it may have been better to use accelerometers to report physical activity; however, for determining whether the children meet or did not meet the Dutch standard for healthy exercise, a questionnaire may be appropriate [85]. “*

Another issue which is really major is that I do not think you have interpreted these results correctly. You conclude that, “our web-based parenting program yields promising effects…” and when I look at your results the ITT analyses (these should be the gold standard) truly show no effects if you do any meaningful correction for multiple comparisons. Again, it looks to me like some of your results are in the wrong direction which also suggests no overall effects. The completers only analyses looks about the same to me. My best guess looking at these results is that this study has more evidence of no effects than of effects, the only place where you see something is the per-protocol analyses but those results are well known to be biased the way you did them.

- *Thank you for this comment and your advice. In the abstract section in line 48-52 we deleted the text “Our results show that the web-based parenting program has promising beneficial effects and can be easily incorporated into existing programs designed to prevent overweight in children.”. And in line 48-54 we added the our new conclusion “Although parents were generally satisfied with the parenting program, following this program had* *no significant beneficial effects regarding the children’s energy balance-related behaviors or the parenting skills compared to the control condition.* *This program may be more beneficial if used by high-risk groups (e.g. parents of children with unhealthy energy balance-related behaviors and/or overweight children) compared to the general population, warranting further study.”. And in the Discussion section in line 747-749 we added the text “Despite the parent’s overall satisfaction regarding the e-learning program and the ease with which our e-learning program can be integrated into the “Scoring for health” program, we found no significant differences between the intervention and control condition.”. In line 760-767 we changed the text into “**Thus, the e-learning program may have beneficial effects if added to an existing overweight-prevention program currently being offered to high-risk groups, for example in the Youth Health Care or in a hospital setting, in which we expect the parents will feel a stronger sense of urgency and therefore be more motivated to use the e-learning program. Thus, future research should test the effectiveness of this e-learning program specifically in high risk groups, such as parents of children with unhealthy EBRBs**, and/or overweight children, thus providing more information regarding the benefits of this program in this specific target population”.*

My final note is that I disagree with the reviewer about dropping the tables with parameter estimates and standard errors, I would focus more rather than less on them.

- *Thank you for this comment and your advice. We replaced table 3-6 because we completely rerun analyses in R**. In our new tables (Table 3 and 4) for the continuous variables we presented the mean and standard deviation at T0, T1 and T2 and the p-values and adjusted p-values. For the dichotomous variables we presented the probabilities and standard deviation at T0, T1 and T2 and the p-values and adjusted p-values.*

*NOTE: In our text we mixed the terms “group” and “condition”, and the terms “modules” and “episodes”. To make te text better readable in lines 155, 208, 209, 218, 219, 232, and 245, and we replaced the terms “control group” and “intervention group” into “control condition” and “intervention condition”, and in lines 230 and 231 we replaced the term “episodes” into “modules”.*

**Journal Requirements:**

When submitting your revision, we need you to address these additional requirements.

1. Please ensure that your manuscript meets PLOS ONE's style requirements, including those for file naming. The PLOS ONE style templates can be found at 
<https://journals.plos.org/plosone/s/file?id=wjVg/PLOSOne_formatting_sample_main_body.pdf> and 
<https://journals.plos.org/plosone/s/file?id=ba62/PLOSOne_formatting_sample_title_authors_affiliations.pdf>

- *We ensure that our manuscript meets PLOS ONE’s style requirements.*

2. Please provide additional details regarding participant consent. In the ethics statement in the Methods and online submission information, please ensure that you have specified what type you obtained (for instance, written or verbal, and if verbal, how it was documented and witnessed). If your study included minors, state whether you obtained consent from parents or guardians. If the need for consent was waived by the ethics committee, please include this information.

- *In our published protocol paper the following text included “We asked the principals’ permission to distribute envelopes to the parents of the children in the participating schools. The envelopes contained an invitation letter in which we asked parents to participate with their child in our study. In addition, the envelopes contained information regarding the study (including the purpose of the study, length of the study, frequency of measurements, eligibility criteria, confidentially of the data, etc.), a passive informed consent form for the parent, a passive informed consent form for the child, and an envelope for returning the forms. Two weeks after distributing the invitation letters, we visited all of the school classes.”.*
- *In the Method section in line 173-176 we added the text “All parents and children received written information about the study [40]. In addition, one researcher (author ER) was present at all classes to provide verbal information regarding the study and answer any questions raised by the children. For this study, we used passive parental consent, which was approved by the Medical Review Ethics Committee.”.*

3. Please provide the original, full-length study protocol, or project proposal, that you submitted to your ethics committee for review and approval prior to study commencement. This document should typically contain a background section, detailed description of the methods used (including information about dates of recruitment, participant characteristics, inclusion/exclusion criteria, detailed descriptions of the protocols/interventions utilized, plans for outcome assessment and data collection/analysis, and references). Please note that we require this to be submitted with your manuscript in both the original language version and a version translated into English.

- *We uploaded our original project proposal in the Dutch language, called “METC_ELVO C1 Onderzoeksprotocol” and the English translation can be found in our published protocol named “The effectiveness of a web-based Dutch parenting program to prevent overweight in children 9–13 years of age: study protocol for a two-armed cluster randomized controlled trial”.*

4. Thank you for submitting your clinical trial to PLOS ONE and for providing the name of the registry and the registration number. The information in the registry entry suggests that your trial was registered after patient recruitment began. PLOS ONE strongly encourages authors to register all trials before recruiting the first participant in a study.

As per the journal’s editorial policy, please include in the Methods section of your paper:

a) your reasons for your delay in registering this study (after enrolment of participants started);

- *We sent the questionnaires to parents after registration in the trial register and after permission from the medical ethics committee, but we have already approached school directors and the program manager of the existing school-based overweight prevention program “Scoring for Health” for possible participation. So that we could start immediately after approval*. It was necessary to have their cooperation before we could submit the study design.

b) confirmation that all related trials are registered by stating: “The authors confirm that all ongoing and related trials for this drug/intervention are registered

- *In the Method section in line 165-166 we added the following text “The authors confirm that all ongoing and related trials for this intervention are registered.”*

5. Thank you for stating the following in the Acknowledgments Section of your manuscript:

"This study was funded by a grant from the Netherlands Organization for Health Research and Development (ZonMw; project number 505010296015), which played no role in the design of execution of this study, the analysis or interpretation of the data, or the decision to publish the results."

We note that you have provided funding information that is not currently declared in your Funding Statement. However, funding information should not appear in the Acknowledgments section or other areas of your manuscript. We will only publish funding information present in the Funding Statement section of the online submission form. 
Please remove any funding-related text from the manuscript and let us know how you would like to update your Funding Statement. Currently, your Funding Statement reads as follows:

"This study was funded by a grant from the Netherlands Organization for Health Research and Development (ZonMw; project number 505010296015, 200100001). This funder had no role in study design, data collection and analysis, interpretation of the data, decision to publish the results, or preparation of the manuscript."

Please include your amended statements within your cover letter; we will change the online submission form on your behalf.

- *No amended statement is required in our online Funding Statement.*
- *In the Declaration section in line 784-788-we deleted the following text: “Funding This study was funded by a grant from the Netherlands Organization for Health Research and Development (ZonMw; project number 505010296015, 200100001). This funder had no role in study design, data collection and analysis, interpretation of the data, decision to publish the results, or preparation of the manuscript.”*
- *In the Declaration section in line 791-794 we deleted the text: “This study was funded by a grant from the Netherlands Organization for Health Research and Development (ZonMw; project number 505010296015), which played no role in the design of execution of this study, the analysis or interpretation of the data, or the decision to publish the results.”.*
- *“No amended statement is required in our Funding Statement.”*

6. We note that you have indicated that data from this study are available upon request. PLOS only allows data to be available upon request if there are legal or ethical restrictions on sharing data publicly. For information on unacceptable data access restrictions, please see <http://journals.plos.org/plosone/s/data-availability#loc-unacceptable-data-access-restrictions>.

In your revised cover letter, please address the following prompts:

a) If there are ethical or legal restrictions on sharing a de-identified data set, please explain them in detail (e.g., data contain potentially identifying or sensitive patient information) and who has imposed them (e.g., an ethics committee). Please also provide contact information for a data access committee, ethics committee, or other institutional body to which data requests may be sent.

- *There are no ethical or legal restrictions on sharing a de-identified data set.*

b) If there are no restrictions, please upload the minimal anonymized data set necessary to replicate your study findings as either Supporting Information files or to a stable, public repository and provide us with the relevant URLs, DOIs, or accession numbers. Please see <http://www.bmj.com/content/340/bmj.c181.long> for guidelines on how to de-identify and prepare clinical data for publication. For a list of acceptable repositories, please see <http://journals.plos.org/plosone/s/data-availability#loc-recommended-repositories>.

We will update your Data Availability statement on your behalf to reflect the information you provide.

- There are no ethical or legal restrictions on sharing a de-identified data set. We uploaded the minimal anonymized data set necessary to replicate our study findings to a repository of the Radboud University in the Netherlands, see <URL>.
- As soon as we have received the URL link we will forward it.

[Note: HTML markup is below. Please do not edit.]

**Reviewers' comments:

Reviewer's Responses to Questions**

**Comments to the Author**

1. Is the manuscript technically sound, and do the data support the conclusions?

The manuscript must describe a technically sound piece of scientific research with data that supports the conclusions. Experiments must have been conducted rigorously, with appropriate controls, replication, and sample sizes. The conclusions must be drawn appropriately based on the data presented.

Reviewer #1: Partly

2. Has the statistical analysis been performed appropriately and rigorously?

Reviewer #1: No

- *Based on the comments of the statistical reviewer and Editor, we revised our strategy for analyses and redid our analyses in the statistical package R. We described our revised strategy for analyses in the Statistical analyses section in line 374-419 , see above the full revised text in the answer to question 3 of the Academic Editor.*

3. Have the authors made all data underlying the findings in their manuscript fully available?

The PLOS Data policy requires authors to make all data underlying the findings described in their manuscript fully available without restriction, with rare exception (please refer to the Data Availability Statement in the manuscript PDF file). The data should be provided as part of the manuscript or its supporting information, or deposited to a public repository. For example, in addition to summary statistics, the data points behind means, medians and variance measures should be available. If there are restrictions on publicly sharing data—e.g. participant privacy or use of data from a third party—those must be specified.

Reviewer #1: Yes

4. Is the manuscript presented in an intelligible fashion and written in standard English?

PLOS ONE does not copyedit accepted manuscripts, so the language in submitted articles must be clear, correct, and unambiguous. Any typographical or grammatical errors should be corrected at revision, so please note any specific errors here.

Reviewer #1: Yes

5. Review Comments to the Author

Please use the space provided to explain your answers to the questions above. You may also include additional comments for the author, including concerns about dual publication, research ethics, or publication ethics. (Please upload your review as an attachment if it exceeds 20,000 characters)

Reviewer #1: Sample size: why the intra class correlation is set at a low level of 0.05?

- *Thank you for this comment. We assume an intraclass correlation coefficient (ICC) of 0.05. This estimate is based on a study on ICCs for obesity indicators in primary schools (Gray et al., 2016). The ICC’s for most outcomes varied between 0.02 and 0.05. Based on this study we decided to use an ICC of 0.05 for the power analysis.*
- *In the method section in line 214-215 we deleted the text: “(with an estimated intraclass correlation coefficient of 0.05 and an average cluster size of 14).”. and in line 215-217 we added the text “We assume an average cluster size of 14 and an intraclass correlation coefficient (ICC) of 0.05* *based on a study on ICCs for obesity indicators in primary schools in which the ICCs for most outcomes were between 0.02 and o.05 [47].”.*

Missing data: what method was used for imputation? There are discrepancies between ITT and per-protocol analysis. It is better to try different method of imputation and evaluate the consistency. Did you consider Race and overweight which are significantly correlated with loss to follow-up?

- *Thank you for this question. We replaced our current analyses in Mplus with analyses with linear mixed models (as recommended by you and the Academic Editor) in R, that more appropriately handles missing data at random. We rewrote our Statistical analyses section to make this clearer. In the method section in line 379-389 we deleted the text about the analysis in Mplus and replaced this text in line 374-379 by “To determine the effectiveness of the intervention, we used linear mixed models in R [66], instead of the planned regression analyses in Mplus as described in the study protocol [40]. Linear mixed models were chosen for two reasons: i) because of the nested data structure of the data (i.e., repeated measurements nested within participants, and participants nested in schools), and ii) because multi-level analyses do not delete participants listwise, and can more appropriately handle data missing at random [67, 68].”*

Data analysis:
Not sure what “multilevel multiple regression” is. It seems that it is better to use generalized linear mixed models (GLMM) for the clustered and repeated dichotomous measure. In the GLMM, the random cluster and time should be tested for significant. The time * intervention interaction should also be tested.

- *Thank you for this comment.* *We replaced our current analyses in Mplus type=complex with the multilevel models recommended by you and the Academic Editor. For this reasons we rewrote our Statistical analyses section. In the method section in line 379-389 we deleted the text about the analysis in Mplus and replaced this text by: “Linear mixed models for continuous outcomes and generalized linear mixed models with logit links for binary outcomes were used. The package lme4 was used for both LMMs and GLMMs [69]. All models included a random effect for students to account for repeated measurements within participants. For each outcome, it was also tested whether it is necessary to additionally account for nesting of students within schools. This additional random factor was only included if the intraclass correlation for the school-level was higher than .05, suggesting significant clustering of students within schools [70, 71]. In addition, it was tested whether a random intercept and slope model significantly better fit the data than the more parsimonious random intercept model using log-likelihood ratio tests. If this was the case, the model with random slopes was used. If the more complex models including additional random factors and/or random slopes did not converge or were singular, we used a more parsimonious model, for example by only modelling random intercepts. Time, condition and their higher-order interactions were included as fixed effects in all models. An unstructured covariance structure was used for all models. Because there were two possible starting points of the study, namely i) February 2013, in which participants of 9 schools were randomized, and ii) September 2013, in which participants of another 2 schools were randomized, the variable ‘moment of randomization’ was included as covariate to correct for possible seasonal effects. To adjust for multiple testing, the p-values of the time x condition interaction effects were adjusted using Holm-Bonferroni sequential correction. All models were visually inspected for homogeneity and normal distribution of errors using residuals versus fitted value plots and Q-Q plots. If it was suspected that these assumptions might have been violated, or if a continuous outcome was strongly left or right skewed, we additionally fitted the models with beta-regression using the glmmTMB package [68]. The results from the beta-regression models were then compared with linear mixed models. Since the outcomes from the linear beta models did not lead to substantially different conclusions, only linear mixed models were reported for continuous outcomes. To determine treatment effects at each of the follow-up timepoints, pairwise post-hoc comparisons of estimated marginal means by time and condition were performed using the emmeans package. For paiwise comparison, p-values were adjusted using the Tukey-method to account for multiple testing. An α of 0.05 was used to indicate statistical significance for all analyses.”*

What baseline characteristics were adjusted in the model? How did you adjust for Race and overweight which are significantly correlated with loss to follow-up? This needs careful consideration and discussion.

- *Thank you for these questions. No baseline characteristics were adjusted in the model. We found no difference between the intervention and control conditions at baseline regarding Race (Ethnicity) and overweight. As reported in our protocol paper, for that reasons we didn’t adjust for these characteristics. However, if desired, we could also additionally check whether including these covariates substantially changes the conclusions drawn from the models.*

If multiple children in the same family were recruited, was the correlation within the same family adjusted in your model?

- *Thank you for this question. See in the method section in line 207-209: “If more than one child living in the same household participated in the study, all participating children in that household were assigned to the same condition as the oldest participating child in order to avoid contamination between conditions.”.*

For so many outcomes, p values need to be adjusted for multiple tests.

- *Thank you for this comment. Based on your comment and the same comment of the academic editor, we now corrected for multiple testing. P-values of interaction effects were corrected using Holm-Bonferroni correction. Post-hoc pairwise comparisons were adjusted using Tukey-method to adjust for multiple testing. Accordingly, in the method section in line 408-409 we added the text: “To adjust for multiple testing, the p-values of the time x condition interaction effects were adjusted using Holm-Bonferroni sequential correction.” In the method section in line 418-419 we added the text: “For pairwise comparison, p-values were adjusted using the Tukey-method to adjust for multiple testing.”*
- *In the Discussion section in line 709-721 we deleted the text:* *“First, it is worth noting that we did not apply Bonferroni correction for multiple testing, as this was the first study to examine the effects of an e-learning program for parents. In this exploratory study, we examined the effects of all relevant variables for which we expected the e-learning program to have an effect. Moreover, we did not expect the program to have particularly large effects, and we did not want to prematurely conclude that the e-learning program did not have an effect. Furthermore, we anticipated wide variations with respect to the outcome measures, as the e-learning program could have had affected slightly different variables among the children and parents (for example, the consumption of SSBs but not fruit could have been affected in one child, while the amount of screen time could have been affected in another child). Thus, applying Bonferroni correction may have been overly strict, as this conservative method can obscure significant results unless the effects and/or sample sizes are extremely large. Furthermore, this approach can increase the likelihood of a type II error, rendering a bona fide difference non-significant [69].”*

Results:
Table 1: separate overweight and obesity

- *Thank you for this suggestion. In the Result section in line 451 in table 1 we now separate overweight and obesity.*

Table 3 can be omitted.

- *Thank you for this suggestion. We omitted table 3 in the main manuscript.* *Because we replaced our current analyses in Mplus type=complex with linear mixed models recommended by you and the Academic Editor.*

Add the p values to Table 4.

- *Thank you for this suggestion. Since we reran all analyses we now report different statistics in Table 3 and 4. In our new tables (Table 3 and 4) we now present the p-values and adjusted p-values for interaction effects for the continuous variables as well as for the dichotomous variables.*

Similarly, for table 5 report OR, 95% CI and p values. Do not report regression coefficients.

- *Thank you for this suggestion. In our new tables (Table 3 and 4) for continuous variables we now present the means and standard errors at T0, T1 and T2 and the p-values and adjusted p-values* *for the interaction effects from linear mixed models. For the dichotomous variables we now present the observed probabilities and standard deviation at T0, T1 and T2 and the p-values and adjusted p-values for the interaction effects from generalized linear mixed models.*

Add p values to Table 6.

- *Thank you for this suggestion. As explained above, In our new tables (Table 3 and 4) we presented the p-values and adjusted p-values for the continuous variables as well as for the dichotomous variables.*

Needs more consideration and discussion about the discrepancies between ITT and per-protocol results.

- *Thank you for this comment. On advice of the Academic Editor we deleted the per-protocol results, because these were not described in our study protocol.*
