# Supplementary material for: The effectiveness of a web-based Dutch parenting program to prevent overweight in children 9–13 years of age: Results of a two-armed cluster randomized controlled trial
Source: PLoS One. 2022 Oct 21;17(10):e0276168. doi: 10.1371/journal.pone.0276168 (PMC9586369; doi:10.1371/journal.pone.0276168)
Supplement: Attachment — Submitted filename: 20220722 Response to Reviewers.docx [file pone.0276168.s007.docx]

**Response to Reviewers**

**Response to Reviewer #1**

Thank you very much for your comment that all comments in our previous revised version have been addressed.

**Response to Reviewer #2**

**Review #2 Comments to the Author**
Thank you for the opportunity to review a revised version of the manuscript, “The effectiveness of a web-based Dutch parenting program to prevent overweight in children 9-13 years of age: results of a two-armed cluster randomized controlled trial.” This paper presents the results of a cluster-randomized trial conducted in the Netherlands of a web-based parenting program known as “Making a healthy deal with your child.” The intervention consisted of five 30-minute modules that walked parents through challenging parenting situations specific to youth’s healthy lifestyle behaviors (e.g., struggles with child consuming vegetables, having excess screen time). While involving parents in childhood obesity-related interventions is the gold standard for treatment, school-based approaches are more common for prevention, with parent involvement and participation generally being more variable in these interventions. In this study, authors ask an important research question in that they aim to assess the value of adding an online self-guided parenting component to an existing school-based intervention, “Scoring for Health.” From my perspective, the authors were quite responsive to the comments of the Editor and statistical reviewer; I defer to them on the need for authors to make further analysis-related revisions.

The authors report on a large clinical trial that was registered and for which a protocol paper has been previously published. Further, authors describe the theoretical underpinnings of the program as coming from strong evidence-based positive parenting programs – parent effectiveness training and the Parent Management Training – Oregon Model. The authors report that they developed the intervention with qualitative input from mothers who lived in low-SES neighborhoods. The study team collected data at three time points, and participant retention rates were good, with 72% completing the 12-month follow up assessment. Authors also reported process evaluation data related to parents’ use of the intervention (e.g., log-in activity, module completion). My concerns include the use of a within-school rather than between-school design due to contamination effects as well as the exclusive use of self-reported measures to assess study outcomes (though the authors do mention both of these in their limitations section). Overall, I think this manuscript will add to the ongoing conversation in the literature about ways to involve parents in obesity prevention efforts using digital approaches. My specific recommendations regarding each section of the paper are detailed below. I hope the authors find these comments useful as they continue to move this work forward.

Abstract:
• Minor - The abstract entered in the manuscript submission portal does not match the abstract in the revised version of the manuscript.

Thank you for your attentiveness. When submitting the revised version, I will ensure that the correct abstract text is stated.

Introduction:
• I encourage the authors to use people-first language when referring to children with overweight or obesity in the introduction and throughout the manuscript. Information and examples of people-first language specific to obesity may be found here: <https://www.obesityaction.org/action-through-advocacy/weight-bias/people-first-language/> For example, rather than using the term “obese children,” use “children with obesity.”

Thank you very much for sharing this knowledge. We would like to contribute to the use of People-first language. For that reason, we have changed “*overweight child*” into “*child with overweight*” throughout the manuscript.

In the abstract section in line 46 we changed “*overweight children”* into *“children with overweight”*.

In the introduction section in line 58 we changed *“overweight and obese children”* into “*children with overweight and obesity”*. In line 59-61 we changed *“In addition, families with low socio-economic status (SES) and families of Turkish and Moroccan descent have a particularly high prevalence of overweight children in the Netherlands”* into *“In addition, in the Netherlands families with low socio-economic status (SES) and families of Turkish and Moroccan descent have a particularly high prevalence of children with overweight”.*

In the discussion section in line 547 we changed *“overweight and/or obese children”* into *“children with overweight and/or obesity”*. In line 550 and line 668 we changed *“overweight children”* into *“children with overweight”*.

• Authors may want to cite some more up-to-date review papers when discussing the role of parents in obesity prevention and treatment more broadly:
o St. George et al. (2020). A developmental cascade perspective of paediatric obesity: A systematic review of preventive interventions from infancy through late adolescence. Obesity Reviews, 21(2), e12939.
o Tomayko et al. (2021). Parent involvement in diet or physical activity interventions to treat or prevent childhood obesity: An umbrella review. Nutrients, 13(9), 3227.
And importantly, include a bit more in the way of parent involvement in digital lifestyle interventions:
o Hammersley ML, Jones RA, Okely AD. Parent-focused childhood and adolescent overweight and obesity eHealth interventions: a systematic review and meta-analysis. Journal of Medical Internet Research. 2016;18(7):e5893.
o Kemp BJ, Thompson DR, Watson CJ, McGuigan K, Woodside JV, Ski CF. Effectiveness of family-based eHealth interventions in cardiovascular disease risk reduction: A systematic review. Preventive Medicine. 2021:106608.

Thank you very much for the more up-to-date review papers, this really helps in discussing the role of parents in obesity prevention and treatment more broadly.

We inserted the reference of the review of St. George et al. (2020) in the introduction section in line 74 and 80, and the review of Tomayko et al. in line 77. In the discussion section in line 551-555 we added the text “*However, systematic reviews by both Bleich et al. and Wang et al. showed that the most promising interventions are delivered in school settings that include a home component [82, 83*]. *In addition, based on their review, Tomayko et al.’s findings support including a parental component in school-based treatment and prevention programs designed to improve the child’s weight and/or weight status outcome [23].*”.

23	Tomayko, E.J., et al., *Parent involvement in diet or physical activity interventions to treat or prevent childhood obesity: An umbrella review.* Nutrients, 2021. **13**(9): p. 3227.

82.	Bleich, S.N., et al., Interventions to prevent global childhood overweight and obesity: a systematic review. The Lancet Diabetes & Endocrinology, 2018. 6(4): p. 332-346.

83.	Wang, Y., et al., What childhood obesity prevention programmes work? A systematic review and meta‐analysis. Obesity reviews, 2015. 16(7): p. 547-565.

We inserted the reference you suggest, namely the systematic review of Hammersley et al, and Kemp et al. in the introduction section in line 101. And in line 99-101 we added the text “*Moreover, systematic reviews have shown that family-based eHealth interventions may be promising as part of a primary prevention-based strategy designed to improve children’s health [39, 40*].”.

• The authors do a nice job distinguising general positive parenting practices from domain-specific positive parenting practices and their relevance to childhood obesity (there is helpful discussion on the distinction between these in a paper by Power, Sleddens, and colleagues (2013) published in Childhood Obesity – Contemporary research on parenting: Conceptual, methodological, and translational issues). However, it is not clear from the hypotheses on page 7 what specific EBRB parenting practices they will examine as secondary outcomes. Please list them here.

Thank you for your advice. To clarify the hypothesis, we have added what specific EBRB parenting practices we will examine as secondary outcomes. In the introduction section in line 151-152 we added the text “*(monitoring, modeling, and applying rules regarding EBRBs)*”. The text in line 150-152 now reads as “*The secondary objectives of the e-learning program include strengthening parenting styles, improving specific parenting practices (monitoring, modeling, and applying rules regarding EBRBs), and increasing parental self-efficacy.*”.

Minor:
• Remove the word “becoming” from the first sentence of the paper. Overweight and obesity in children is a major health concern.

Thank you for this comment. As you suggested in the introduction section in line 58 we removed the word “*becoming*”.

• Missing the word “time” in the following sentence on page 6: “v) children spending an excessive amount of [time] using the computer and not wanting …”

Thank you for your attentiveness. As you suggested in the introduction section in line 132 we added the word “*time*”.

Methods and Results: These sections seem to have greatly benefitted from responding to the suggestions of the Editor and statistical reviewer. I have only a few questions/comments:
1. It appears as though parents assigned to the intervention condition received an email up front with their log-in code/access to the modules and then a congratulatory email following the completion of each module. I was less clear as to whether there were any automated reminder emails or any follow-up from study staff encouraging parents to complete the modules --- this information is included in the discussion but might be helpful to note in the methods section.

Thank you for this comment. All parents who were allocated to the intervention condition received an email with their log-in code/access to the modules and then a congratulatory email following the completion of each module in this email the parents was also encouraged to complete the next module. In addition, the study staff send two reminder e-mails to the parents asking them to complete the e-learning program.

For clarification and to make the text more readable, in the methods section 228-229 we replaced the text “*The parents in the control condition received only the brochure.*” to line 222-223. Next, in line 224 we added the text “*in the intervention condition”*. Last, we have added the following text in line 226-228 “*In addition, we sent reminder e-mails 7 and 12 weeks after providing the log-in code, reminding parents to complete the e-learning program.*”.

2. Authors currently present process data as parents who completed one module or 2 or more modules. How many parents completed 3, 4, and 5 modules? I was interested in more granular details regarding module completion as I believe this may have important implications for understanding study findings.

Thank you for this question.

As mentioned in the result section in line 496-501: In the intention-to-treat framework, 171 (68%) of the 252 parent-child dyads in the intervention condition actually started the e-learning program; 109 (64%) of these parents completed 2 or more modules, and 85 parents (50 %) completed all 5 modules in the e-learning program (dose received). In addition, 81 (32%) parent-child dyads of the 252 parent-child dyads in the intervention condition did not start the e-learning program.

Below you can see how many parents completed 1, 2, 3, 4, and 5 modules of the total of 252 parent-child dyads in the intervention condition. The percentage of number of parents in the table below are the percentages of the total of 252 parent-child dyads in the intervention group.

It is important to know that the group of parent-child dyads which completed 0 episodes (n=122), consist of i) parents who did not start the e-learning (n=81), and ii) parents who start the e-learning but did not finish any episode (n=41).

| **Number of episodes completed**  **(N)** | **Number of parents of the intervention group**  **(%)** |
| --- | --- |
| 0 episodes | 122 (48.4) |
| 1 episode or more | 130 (51.6) |
| 2 episodes or more | 109 (43.3) |
| 3 episodes ore more | 92 (36.5) |
| 4 episodes or more | 88 (34.9) |
| 5 episodes or more | 85 (33.7) |

In the result section in line 497 we replaced (68%) to line 496 to make the text more readable. Additionally, in line 496 we replaced “*framework*” into “*approach*”, in line 497 we added “*109*” and “*171*” and in line 498 we added *“64%”* and the text “and 85 parents (50%) completed all 5 modules”. The text in line 496-499 now reads as: *“In the intention-to-treat approach, 171 of the 252 parent-child dyads (68%) in the intervention condition started the e-learning program; 109 of these 171 parents (64%) completed 2 or more modules, while 85 of these parents (50%) completed all 5 modules of the e-learning program.”*

In Table 5 in S5 Appendix we added an extra column “completed all 5 modules”. The appendix is now named as “S5 Appendix_revised”.

3. It’s interesting that measures of height were taken using a tape measure rather than a stadiometer. Why was this instrument selected to assess height (maybe ease of transporting)?

A tape measure was used because this instrument was at that time used by the Youth health care department of the community health services, also because of the ease of transporting.

4. Where the response options for the parental EBRB rules measure (i.e., strict, indulgent, no rules) part of the original measure or did the authors develop these for the current study? I wondered why an option around the presence of rules that were reasonable (somewhere in between strict and indulgent) was not included.

Thank you for this question. The 3 response options for parental EBRB rules (strict, indulgent, no rules) were taken exactly from the original measure. The question about parental EBRB rules is derived from the Child Monitor questionnaire. This Child Monitor questionnaire is part of the "Local and National Health Monitor" in the Netherlands, which uses standard questions. The Child Monitor is part of a monitoring cycle and is required by the Dutch Public Health Act. Each four years a cross-sectional survey is conducted to gain insight into the population’s health, lifestyle and well-being of children age 6 months to 12 years [1] [2, 3]. The questions in the Child Monitor questionnaire about establishing EBRBs rules were only for parents of children 4-12 years of age.

1.	Van der Star, M., *Kindermonitor 2010: Gezondheidsonderzoek kinderen 0-12 jaar regio Nijmegen [Child Monitor 2010: Health research in children 0-12 years of age in the Nijmegen region, the Netherlands]*. 2010, GGD Nijmegen: Nijmegen.

2.	Van der Star, M., *Regiorapport Kindermonitor 2009/2010: Gezondheid, welzijn en leefwijze van 0-12 jarigen in de regio Nijmegen. [Region report Child Monitor 2009/2010: Health, well-being and lifestyle of children 0 to 12 years of age in the Nijmegen region]*. 2010, GGD Gelderland-Zuid: Nijmegen.

3.	Ruiter, E., et al., *Parents' underestimation of their child's weight status. Moderating factors and change over time: A cross-sectional study.* PloS one, 2020. **15**(1): p. e0227761.

To clarify, in the method section in line 321-324 we added the text “*Parental EBRB rules were assessed via the parental and child questionnaire. Seven items were derived from the Child Monitor Questionnaire, which is part of the "Local and National Health Monitor" in the Netherlands [57]. Children and parents were asked*…”. In line 330-332 we added text with the brief three definitions of the three categories. The text now reads as: *“**The children and parents were instructed to answer each question with the following possible answers: ‘i) yes, and we stick to them (“strict rules”); ii) yes, but we are flexible with them (“indulgent rules”), or iii) no, we have no rules about it (“no rules”).”.*

Discussion:
• Authors note “our findings indicate that the program has room for improvement, as the intervention was not used intensively enough.” I think this point may be understated and that authors could discuss the issue of dose received in more detail earlier in the discussion rather than waiting until the strengths/limitations section. It is difficult to ascertain whether or not the intervention ‘worked’ when uptake was low.

Thank you for this comment and your advice. We discussed *“*our findings indicate that the program has room for improvement, as the intervention was not used intensively enough.” before the strengths and limitation section, namely in line number 555-556. However, we agree that we can discuss the issue of dose received in more detail as you advised. In the method section in line 341-342 we added the text *“(dose received) of the in total 5 modules delivered in the e-learning program (dose delivery)*.”. The text in line 340-342 now reads as “*This approach also provided information regarding the number of modules each parent completed (dose received) of the in total 5 modules delivered in the e-learning program (dose delivery).*”. In the result section in line 499 we added the text *“(dose received)*”, the text in line 496-499 now reads as “*In the intention-to-treat approach, 171 of the 252 parent-child dyads (68%) in the intervention condition actually started the e-learning program; 109 of these 171 parents (64%) completed 2 or more modules, and 85 of these parents (50 %) completed all 5 modules of the e-learning program (dose received).*”. In the discussion section in line 556-557 we changed the text *“The intensity of the program is also a serious point that warrant discussion.”* into *“The intensity at which the parents in our study were exposed to the intervention also warrants discussion.”*. Next, in line 557-569 we added the text “*We found that 64% of parents in the intervention condition who started the e-learning program completed 2 or more modules, with 50% of parents completing all 5 modules (the total delivery dose). However, if we consider all 252 parent-child dyads in the intervention condition (i.e., both the group of parents who started the program and the group of parents who never started the program), only 43% and 34% of all parents assigned to the intervention condition completed 2 or more modules or all 5 modules, respectively. Following 2 or more of the 5 modules is the minimum requirement to be considered sufficiently exposed to the e-learning program. Thus, in our study only 43 % of the total intervention condition was sufficiently exposed to the intervention. Therefore, the relatively low percentage of parents who were sufficiently exposed to the intervention may explain why we found no apparent difference between the intervention and control conditions, making it difficult to determine whether the intervention was actually effective given the high percentage of parents (57%) who were not sufficiently exposed to the intervention.*”.

Last, in line 575 - 576 we deleted the text “*For example, if parents do not feel a sense of urgency to focus on healthy EBRBs, it may not be self-evident to complete all modules.*”, and we replaced the text *“On the other hand, the amount of time parents needed to complete the program is relatively short, with 5 modules lasting approximately 30 minute each; thus, program was not necessarily expected to have a robust effect.”* from line 576-578 to line 645-647.

• Related to my comment above, engagement in eHealth interventions is arguably one of the biggest challenges in the field.

To what extent did authors include their target population in the process of developing their intervention modules?

Thank you for this question. We include our target population in the process of developing our intervention modules by organizing focus group conversations. The aims of this focus group study were to i) explore and identify everyday life situations in which mothers experience difficulties stimulating healthy EBRBs in their school-age child, and ii) identify the reasons why mothers encounter these difficulties. The e-learning program’s content was based on the results of these focus groups [1]. Before testing the e-learning in an RCT, the e-learning was viewed by 5 mothers of different ethnic backgrounds who participated in the focus group study. The e-learning was optimized with the feedback obtained from these mothers.

1. Ruiter, E.L., et al., *Everyday life situations in which mothers experience difficulty stimulating healthy energy balance–related behavior in their school-age children: a focus group study.* BMC public health, 2019. **19**(1): p. 701.

Did authors follow up with families to understand reasons they did or did not view the modules?

Unfortunately, it did not fit within our research project to follow up with families to understand reasons they did or did not view the modules. This would have been meaningful and could be a recommendation for further research.

They speculate that participants may not have been the appropriate targets and that a higher risk group may have been more appropriate, which is a fair point, but other strategies for increasing engagement beyond email reminders may have been helpful. For example, Mohr and colleagues have argued that supportive accountability via human contact may be required to get participants to engage with digital interventions. See: Mohr et al. (2011). Supportive accountability: a model for providing human support to enhance adherence to eHealth interventions. Journal of Medical Internet Research, 13(1), e30)

Thank you for this comment. In the discussion section in line 578-603 we added the text “*In addition, we used a relatively minimal engagement strategy. Our goal was to develop a self-guided web-based parenting program that can be added easily to existing overweight-prevention programs such as existing school-based/population-based interventions, thereby maximizing integration and minimizing costs. In one respect, this minimal-engagement strategy was successful, as 68% of parents started the intervention, with 64% of them completing at least 2 of the 5 modules, and 50% completing all 5 modules. Unfortunately, despite this relatively high level of involvement we found that the intervention had no apparent effects on either parenting skills or the children’s EBRBs. Using additional strategies to increase engagement in addition to sending two email reminders may have increased the percentage of parents who participated in the intervention, thus increasing the likelihood of revealing an intervention effect. In their supportive accountability model, Mohr et al. argue that human support increases adherence by increasing accountability to a coach who is seen as trustworthy, benevolent, and having expertise, moderated by motivational factors such as the communication media used (e.g., face-to-face, telephone, or email) [84]. However, we expect that parents of children in a high-risk group will be more willing to change their child’s EBRBs, feel a stronger sense of urgency to change their parenting skills, and would therefore be more motivated to use the e-learning program. We would therefore expect a higher percentage of parents to follow the e-learning program, thus revealing intervention effects. Moreover, we expect to find more room for improvement among the high-risk group with respect to improving parenting skills and their child’s EBRBs. On the other hand, if we target a high-risk group who are currently under the care of a health-care professional, it should be easier to increase engagement, as a trusted health-care professional is already involved and can provide the parents with face-to-face support, increasing their likelihood of starting—and ideally, completing—the e-learning program. This healthcare professional can also make follow-up appointments with the parents, and use this appointment also to discuss whether the e-learning program was completed successfully, or —if necessary—encourage them to complete the e-learning program.*”.
